# Alginate Nanoformulation: Influence of Process and Selected Variables

**DOI:** 10.3390/ph13110335

**Published:** 2020-10-23

**Authors:** Hazem Choukaife, Abd Almonem Doolaanea, Mulham Alfatama

**Affiliations:** 1 Faculty of Pharmacy, Universiti Sultan Zainal Abidin, Besut Campus, Terengganu 22200, Malaysia; hazemchoukaife@gmail.com; 2 Department of Pharmaceutical Technology, Kulliyyah of Pharmacy, International Islamic University Malaysia, Kuantan 25200, Pahang, Malaysia; abdalmonemdoolaanea@yahoo.com

**Keywords:** alginate, nanoparticles, drug delivery, encapsulation, emulsification/gelation, electrospray

## Abstract

Nanocarriers are defined as structures and devices that are constructed using nanomaterials which add functionality to the encapsulants. Being small in size and having a customized surface, improved solubility and multi-functionality, it is envisaged that nanoparticles will continue to create new biomedical applications owing to their stability, solubility, and bioavailability, as well as controlled release of drugs. The type and physiochemical as well as morphological attributes of nanoparticles influence their interaction with living cells and determine the route of administration, clearance, as well as related toxic effects. Over the past decades, biodegradable polymers such as polysaccharides have drowned a great deal of attention in pharmaceutical industry with respect to designing of drug delivery systems. On this note, biodegradable polymeric nanocarrier is deemed to control the release of the drug, stabilize labile molecules from degradation and site-specific drug targeting, with the main aim of reducing the dosing frequency and prolonging the therapeutic outcomes. Thus, it is essential to select the appropriate biopolymer material, e.g., sodium alginate to formulate nanoparticles for controlled drug delivery. Alginate has attracted considerable interest in pharmaceutical and biomedical applications as a matrix material of nanocarriers due to its inherent biological properties, including good biocompatibility and biodegradability. Various techniques have been adopted to synthesize alginate nanoparticles in order to introduce more rational, coherent, efficient and cost-effective properties. This review highlights the most used and recent manufacturing techniques of alginate-based nanoparticulate delivery system, including emulsification/gelation complexation, layer-by-layer, spray drying, electrospray and electrospinning methods. Besides, the effects of the main processing and formulation parameters on alginate nanoparticles are also summarized.

## 1. Introduction

Nanomaterials have recently received much attention as drug carriers. In particular, nanoparticle-based nanoparticles [1,2], lipids [3,4], magnetic nanoparticles [5,6] and liposomes [7] have been widely studied in drug delivery systems. Among all, polymeric nanoparticles have been widely investigated due to their unique physicochemical properties [8]. The natural and synthetic polymers are versatile materials and are preferred for many applications, including the pharmaceutical industry [9].

Naturally-derived polymers are superior to synthetic ones due to their biodegradability, biocompatibility and biological activity. Hydrogel-based natural polymers such as alginate, collagen and gelatin can be used to deliver hydrophilic drugs due to their ability to absorb large amounts of water while maintaining their structures. The water absorption ability is related to the hydrophilic groups such as –OH, –COOH, –CONH –SO_3_H and –CONH_2_ that are contained in the hydrogel matrices [10]. Besides, the chelating, biocompatible, immunogenic and mucoadhesive properties of alginate make it as an attractive polymer in drug and cell delivery systems [11,12]. Alginates are considered among the most biosynthesized polymers, where 70% of annual alginate production is allocated to pharmaceutical and biomedical applications and the remaining is used in the food industry [13,14].

After alginate evolution in the 1980s and their spread as microparticles for encapsulation purposes, several studies were carried out to synthesize nano-sized alginate particles [15,16]. Gelling properties of alginate and its remarkable processing ease have grown its importance in drug delivery, cell immobilization, food industry and research perspectives [17,18]. Furthermore, it is considered as an environmentally friendly polymer that can undergo recycling and degradation. Alginate nanomaterials represent a fast-developing field, particularly for the pharmaceutical and food industry as well as academia. The gelling property makes alginate polymer as one of the most frequently used in drug delivery [19].

The physicochemical properties of alginate, such as viscosity, thermo-stability, sol-gel transformation, pH-responsivity, as well as drug release can gain better insight into their potential applications. Many factors could impact the properties of alginate nanoparticles, such as alginate, surfactant and crosslinker concentrations, stirring time and speed as well as pH value [20]. In this review, different aspects related to the formulation and processing parameters using various techniques for preparation of alginate nanoparticles were discussed with regard to the size, encapsulation efficiency, zeta potential and drug release profile.

## 2. Alginate Polymer

**Figure 1:**
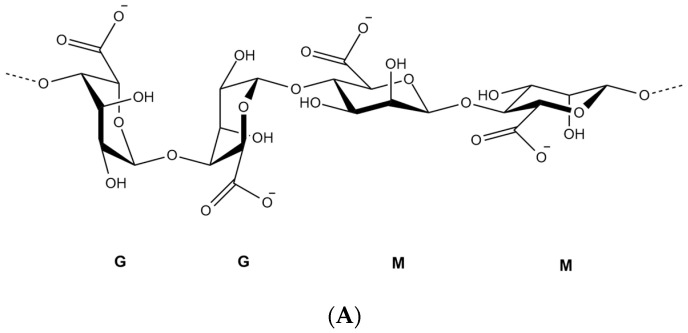

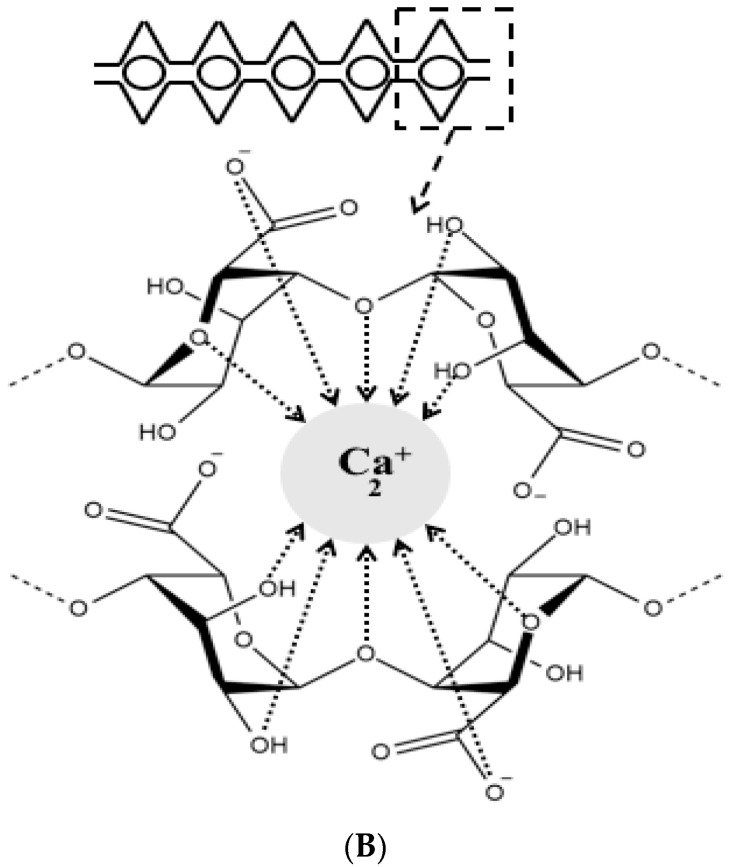
Chemical structure of alginate displaying the d-mannuronate (M) and l-guluronic (G) blocks (**A**), schematic representation of calcium binding to alginate to form an egg-box shape (**B**).

Over the last decades, researchers were extensively utilizing natural polymers, especially in the pharmaceutical [21] and food industry [22,23] due to their advantages such as biocompatibility, biodegradability and low cost [11]. Alginate is an anionic polymer that typically obtained from brown marine algae. It is an unbranched polysaccharide copolymer consisting of alternating of d-mannuronate (M) and l-guluronic (G) blocks linked together by 1,4-glycosidic linkages (Figure 1A). Divalent cations, such as Ba^2+^ and Ca^2+^, can quickly form so-called egg-box complexes with G block to create alginate hydrogel through gelation phenomenon (Figure 1B) [24]. Hydrogelling ability of alginate has broadened its applications in biomedical and pharmaceutical research to encapsulate proteins and drugs for controlled release and targeted delivery. Hydrogel alginate matrix is a pH responsive where it shrinks at low pH, thus, the payload is preserved for extended period of time. Conversely, it swells and releases the encapsulated drug at higher pH values, offering a great carrier for oral delivery.

## 3. Sources of Alginates, Extraction, and Purification Methods

Alginates are unbranched polysaccharide, present in the c + ell walls of brown algae as well as some bacteria such as *Azotobacter* and *Pseudomonas* spp. [25,26]. Typically, the main sources of commercial alginates are from *Laminaria hyperborea*, *Laminaria digitata*, *Macrocystis pyrifera*, *Ascophyllum nodosum*, *Eclonia maxima*, *Laminaria japonica*, *Lessonia nigrescens*, *Durvillea antarctica* and *Sargassum* spp. [27]. Alginate chains are composed of units of β-d-mannuronic acid and α-l-guluronic acid of different arrangement depending on their natural source with pKa range from 3.38 to 3.65. Alginate is precipitated as an insoluble alginic acid in the low pH medium at room temperature [28]. Under alkaline extraction, alginates are purified with sodium hydroxide, sodium carbonate, or gelatinous aluminum hydroxide from the powdered brown algae. After purification, alginates are filtered and subsequently precipitated with Ca^2+^ ions/ethanol or by means of acidification [27,29].

Essentially, in order to avoid a brown discoloration of the final product, depigmentation of algae powder should take place prior to the extraction step. In addition, polyphenols are considered as another impurity that alter the rheological properties of alginate by forming strong dipolar forces with polysaccharides [30]. In order to render polyphenols insoluble during the extraction process, algae powder is soaked in formaldehyde or in a mixture of formaldehyde and ethanol [27]. Finally, polyphenolic levels in the final product are determined using fluorescence spectroscopy at 450 nm wavelength [31].

## 4. Alginate Nanoparticles

**Figure 2:**
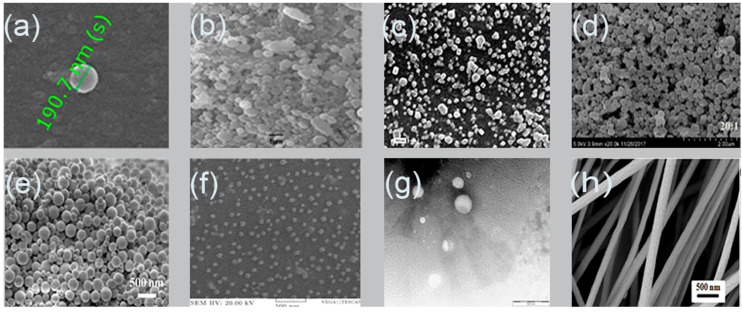
Scanning electron microscopy images of alginate nanoparticles prepared by: (**a**) emulsification/internal gelation, 1% *w*/*w* alginate, CaCO_3_:alginate mass ratios 0.1:1, pH 6 medium chain triglyceride (MCT) oil [40]; (**b**) nanospray dryer, spray cap 7 µm, flow rate 7 mL/min, drying gas flow of 110 L/min with relative flow rate 100%, inlet drying gas temperature 120 °C and outlet temperature 35 °C [41]; (**c**) polyelectrolyte complexation, nisin-loaded nanoparticles alginate 250 mg/mL and chitosan 250 mg/mL [42]; (**d**) evaporation method, zein-to-propylene glycol, alginate mass ratio 20:1 [43]; (**e**) layer-by-layer, paclitaxel, poly (lactic-co-glycolic acid) (PLGA) 10 mg/mL, alginate 5 mg/mL and chitosan 5 mg/mL [44]; (**f**) emulsification/external gelation, alginate 0.03% *w*/*v* and CaCl_2_ 18 mM [45]; (**g**) electrospray DNA plasmid loaded alginate nanoparticles, flow rate 0.1 mL/h, voltage 12.5 kV, alginate 1% *w*/*v*, Tween 20 1% *v*/*v*, CaCl_2_ 1.5% *w*/*v*, collector distance 4 cm and nozzle size 30 G [46]; (**h**) nanofiber produced by electrospinning, alginate 1.74% *w*/*w*, voltage 12 kV, needle 27 G, flow rate 0.6 mL/h and distance 12 cm [47].

The remarkable properties of alginate enable the flexibility to synthesize various designs of particles such as nanoparticles and nanofibers. Nanosystem is a potential tool for controlling drug stability, delivery and release, which may improve drug bioavailability as well as expand the choices of drug administration routes [32]. Nanoparticles or ultrafine particles are defined as solid spheres of size range from 10 to 1000 nm [33]. Pharmaceutically, nanoparticles are made of biocompatible and biodegradable polymers of natural or synthetic origin, in which the pharmaceutical agent can be entrapped in or diffused into the particle matrix during the synthesis process [34]. Nanofibers, on the other hand, possess high surface area, controllable pore structure, diameter less than 1000 nm, and light weight compared to the conventional fibers [35,36]. The noticeable properties of nanofibers render them highly useful in biomedical [37] and drug delivery applications [38] as well as skin tissue engineering [39]. Figure 2 shows images of alginate nanoparticles/nanofibers produced by different fabrication approaches using scanning electron microscope (SEM).

The small size and large surface area of nanoparticles increase the dissolution rate and solubility of poorly soluble drugs. Nanoparticles can enhance the targetability of the encapsulant to specific sites of the body/tissue/cells, whether paracellularly or transcellularly [48]. The relationship between the rate of dissolution and particles size as well as surface area is explained by Noyes–Whitney Equation (1) as follows [49]:

(1) $$\frac{dc}{dt}=\frac{D\;\times \;A\;\left(Cs\;–\;C\right)\;}{h}$$

where *dc*/*dt* = dissolution rate, *D* is the diffusion coefficient of the substance, *A* is the surface area of exposed solid, *Cs* and *C* represent the concentration of the dissolved substance at a given time t and the solubility concentration of the substance, respectively, and *h* is the thickness of the diffusion layer.

Also, nanoparticles enhance the solubility, dissolution rate and bioavailability of poorly soluble drugs by promoting the interaction propensity with the medium, owing to their large surface area [50]. Several researchers had developed and characterized nanoparticles-based natural polymers [51], lipids [52], polysaccharides [53] and synthetic biodegradable polymers over the last ten years [54]. Due to the unique physicochemical properties of alginate polymer among various natural polysaccharides, the ability to encapsulate foods, drugs and proteins into alginate nanoparticles has attracted the interest of many researchers [14]. Alginate-based nanocarrier seems to have all optimal requirements to be a successful drug delivery system due to its biodegradability, biocompatibility, protection effect of oral drugs against harsh gastrointestinal environment, controllable release, water solubility (avoiding the effect of noxious solvents during processing), availability and low cost [55]. Various studies have focused on enhancing the low intestinal penetration, gastrointestinal degradation and low bioavailability of orally administered insulin utilizing alginate nanoparticles as an oral drug delivery system [56,57]. On top of that, the application of alginate nanoparticles in cancer treatment has gained wide attention due to the ability to deliver anti-cancer therapeutics in sufficient manner at target site, promoting the bioavailability as well as reducing drug dosage and its side effects to the normal tissues [58,59]. Alginate nanoparticles have been also used for targeted antibiotic delivery applications without inducing resistant strains of bacteria [60,61]. The applications and fields that utilized alginate nanoparticles grew proportionally due to their useful properties and simple synthesis methods.

## 5. Alginate Nanoparticles Preparation Methods

Alginate nanoparticles are utilized as carriers to improve the bioavailability of drugs. Various techniques have been proposed to synthesize alginate nanosystem. Selection of the preparation method is highly related to the nature of encapsulant, as well as the pre-determined attributes that nanoparticles should meet. The following sections review the most common fabrication methods of alginate nanoparticles and their effective variables.

### 5.1. Emulsification/Gelation

**Figure 3:**
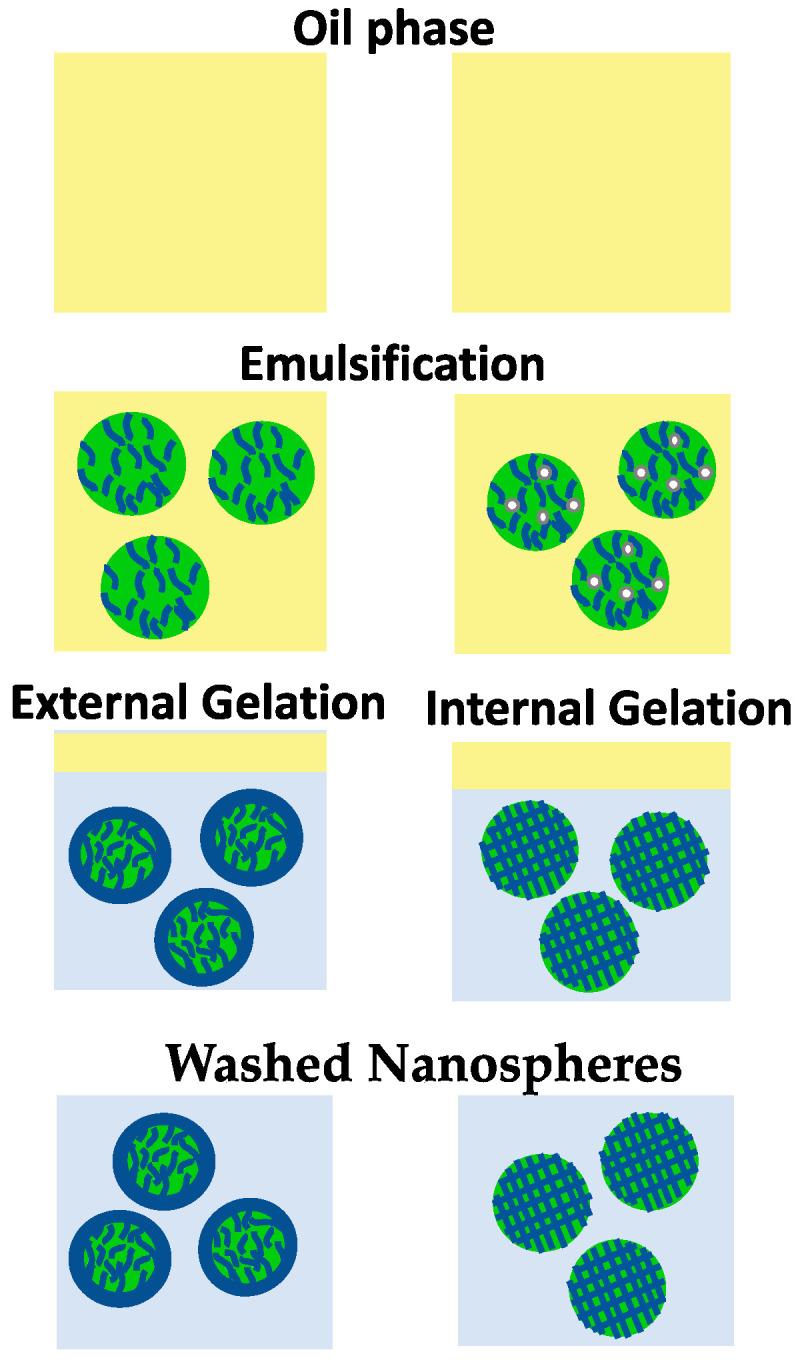
Schematic presentation of alginate nanospheres preparation by means of emulsification (external/internal) gelation method.

Emulsification/gelation is defined as a gelation process of the emulsion droplets that consist of an alginate solution dispersed in an oil phase to fabricate nanospheres [40]. Generally, this is a simple and low-cost technique compared to the nozzle-based methods [62,63]. This technique consists of two main stages: preparation of alginate-in-oil (w/o) emulsion, followed by gelation of the alginate emulsion droplets in aid of a covalent or ionic crosslinker [16]. Gelation of alginate takes place through two conventional ways: external and internal gelation [64]. For external gelation, crosslinker such as CaCl_2_ diffuses from the outer phase into the inner core of alginate emulsion droplets to react immediately with carboxylic groups of α-L-guluronic acid. Typically, after gelation of alginate emulsion droplets by adding the crosslinker, the emulsion is demixed (Figure 3) [16,19]. Emulsification/external gelation outputs micro/nanospheres consisting of a soft core and a rigid outer matrix [65]. Internal gelation technique on the other hand, depends on the release of cations from the inner core of alginate emulsion droplets [66]. Basically, a water-insoluble calcium salt, such as CaCO_3_, is mixed with alginate before emulsification (Figure 3). Gelation of alginate is initiated by increasing the solubility of calcium source and/or lowering the pH of the emulsion from 7.5 to 6.5, where calcium ions begin to migrate from the inner droplets to the outer part as shown in Equations (2) and (3) [67,68]. Internal gelation produces symmetrical micro/nanospheres with large pores and low matrix density compared to that prepared by external gelation [60,61].

2H^+^ + CaCO_3_ → Ca^2+^ + CO_2_ (2)

Ca^2+^ + 2Na^+^Alg^−^ → Ca^2+^(Alg^−^)_2_ + 2Na^+^ (3)

Paques et al., have prepared alginate nanospheres via w/o emulsification followed by internal gelation using CaCO_3_ micro/nano particle. The concentration of alginate solution was at 1% *w*/*w* and CaCO_3_ to alginate weight ratio at 0.1:1. Glucono delta-lactone (GDL) as an acidifier, together with CaCO_3_ were dispersed in alginate solution. The final mixture was subsequently emulsified in the continuous phase of the medium chain triglyceride (MCT) oil. The results showed that gelation time and size of alginate spheres reduced proportionally to CaCO_3_ size from micro to nano-sized, while higher alginate concentration has resulted in delaying of the gelation time. The mass ratios at 0.1:1 and 1.98:1 of CaCO_3_:alginate and GDL:CaCO_3_, respectively, with pH value around 6 were considered as the optimum values in gel properties [40].

Another study has fabricated doxorubicin-loaded alginate nanospheres via w/o emulsification/external gelation intended for breast cancer therapy. The formulation composed of an aqueous phase (doxorubicin dissolved in alginate solution) mixed with an organic phase (cyclohexane/dodecylamine) by stirring at 1200 rpm. The synthesized nanospheres exhibited particle size at 82.8 nm, polydispersity index at 0.204 and zeta potential at +7.2 ± 4.6 mV [69]. Total of 30% of the payload released at pH 5.5 over the course of 4 h followed by 90% released by 24 h at pH 7.4.

In the Spadari et al. study, miltefosine (antifungal drug) has been encapsulated in alginate nanospheres by emulsification/external gelation method. Miltefosine was dissolved in an aqueous alginate solution (1% *w*/*w*) followed by emulsification with sunflower oil (3% *v*/*v*) containing Span 80 as a surfactant. After sonication of the mixture in an ice bath, CaCl_2_ and Poloxamer 407 were added dropwise to the emulsion as a crosslinker and surfactant, respectively. The mixture was sonicated for 5 min, accompanied by stirring for 30 min. Subsequently, the system was centrifuged at 3000× *g* for 5 min, discarding the supernatant followed by oil residues removal through complexation with isopropanol. In order to obtain a homogeneous fine powder of alginate nanospheres, 500 μL of trehalose (10% *w*/*v*) was applied prior to freeze-drying for 24 h. The dried nanospheres had a zeta potential of −39.7 ± 5.2 mV with a mean particle size of 279.1 ± 56.7 nm, a polydispersity index of 0.42 ± 0.15 and an encapsulation efficiency of 81.70 ± 6.64%. The study concluded that emulsification/external gelation method had efficiently encapsulated miltefosine in alginate matrix with an enhanced antifungal effect for treatment of *Galleria mellonella* infection and reduced drug toxicity [70].

### 5.2. Emulsification-Solvent Displacement Technique

Emulsification-solvent displacement method was first described in 1997 when Quintanar et al., proposed a new way to prepare concentrated pseudolatex nanoparticles using acceptable solvents [71]. This process involves emulsifying an organic solution of the polymer and drug (water-saturated) in an aqueous phase of a stabilizer (solvent-saturated) through conventional stirrers, accompanied by direct displacement of solvents under rapid evaporation using vacuum and/or temperature (e.g., rotary evaporator). Compared to conventional solvent evaporation method, emulsion homogenization step is avoided in emulsification-solvent displacement approach to produce nanoparticles. This method depends on the aggregation of the polymer and drug through a rapid diffusion of the solvent from the internal into the external phase [72].

In the study of Dai et al., zein nanoparticles were synthesized through Pickering emulsions of zein and propylene glycol alginate (PGA) via solvent evaporation method. Firstly, PGA was mixed with ethanolic zein solution at various mass ratios of 1:1, 5:1, 10:1, 20:1 and 40:1, then ethanol was evaporated by rotary evaporator followed by freeze drying for 3 days to obtain solid zein-PGA nanoparticles. The results showed that the nanoparticle size was significantly reduced at 10:1 mass ratio, while higher PGA concentration enhanced the sample stability as a function of strong electrostatic repulsion between particles, promoting the production of stable Pickering emulsions [43]. 

### 5.3. Solvent Evaporation Technique

**Figure 4:**
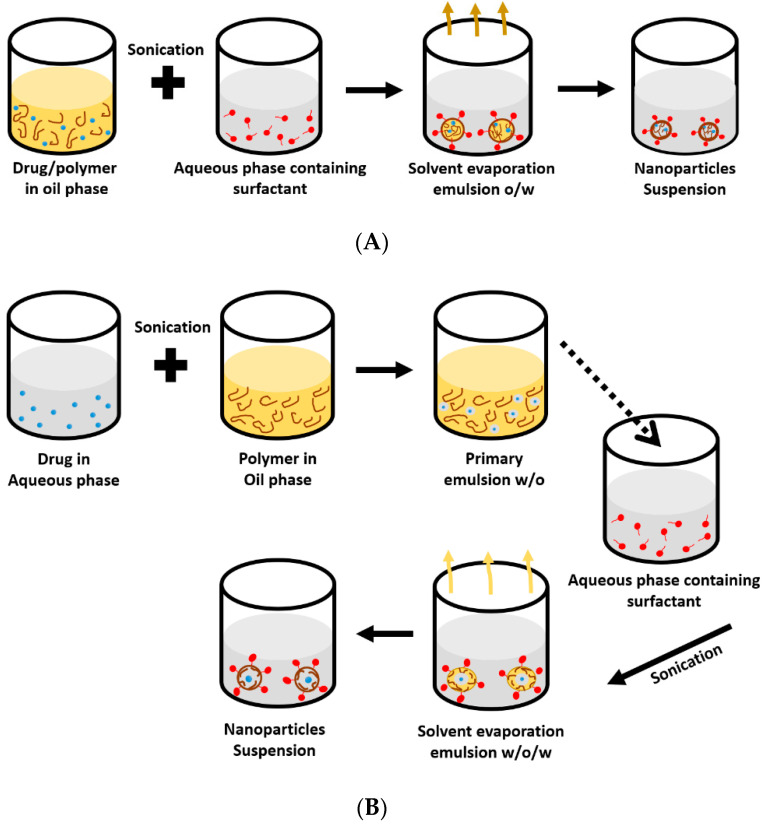
Schematic representation of nanoparticles formulated by single emulsion technique (**A**), and double emulsion technique (**B**).

In this technique, the polymer and the hydrophobic drug are dissolved in a volatile organic solvent [8]. The emulsion (o/w) is prepared by adding organic solution such as ethyl acetate, dichloromethane or chloroform (oil phase) to the aqueous solution of surfactant under ultra-sonication or rapid homogenization [73,74]. The organic solvent is then evaporated by means of continuous stirring or high temperature under reduced pressure. The nanoparticles are then collected through ultracentrifugation to remove the excess solvent, while free drug and surfactant are washed away with distilled water (Figure 4A). Double emulsion (w/o/w) technique on the other hand, is commonly used for encapsulation of hydrophilic molecules such as proteins, peptides and antigens (vaccines), where aqueous drug phase is added to the oil phase of polymer and volatile solvent under continuous stirring to prepare stable emulsion (w/o). The resulted emulsion is then transferred under the same condition to the aqueous phase of surfactant to produce a double emulsion (w/o/w) (Figure 4B) [8]. Under the same condition, nanoparticles are hardened after solvent removal, while the physical and chemical properties of the synthesized nanoparticle can be tuned by tailoring the emulsion properties, such as surfactant concentration/type, o/w phase ratio, polymer concentration and evaporation rate as well as processing parameters, including agitation rate and time, geometry of homogenizing tip, and shape and volume of homogenizer vessel [75,76].

In the study of K.S et al., zidovudine nanoparticles were prepared from alginate and stearic acid polyethylene glycol via two-step emulsion solvent evaporation technique. This study aimed to confer enhancement of the drug loading capacity, the drug release efficacy and the biocompatibility. The organic phase consisted of a chloroform as a solvent, polyethylene glycol (0.2% *w*/*v*) and stearic acid (100 mg in 25 mL), while zidovudine was dissolved into the alginate solution (1 mg/mL) to form the aqueous solution. The first emulsion was prepared by dissolving polyethylene glycol (0.2% *w*/*v*) and stearic acid (0.4% *w*/*v*) in chloroform at 55 °C. Zidovudine of different strengths (100 mg, 150 mg and 200 mg) was dissolved in alginate (0.1% *w*/*v*) solution at 55 °C to form a double emulsion system, stirred for 2 h in a fume hood to evaporate the organic solvent and solidify the nanoparticles. Then, nanoparticles suspension was centrifuged (20,000 rpm), washed and dried by freeze-drying technique. The optimized formulation produced particles size of 407.67 ± 19.18 nm, zeta potential of −42.53 mV and encapsulation efficiency of 83.18 ± 22%. The drug release was examined in different dissolution medium at pH 1.2 and 7.4. Initially, zidovudine nanoparticles exhibited a burst release around 36% and 20% at pH 1 and 7.4, respectively within 2 h, followed by a prolonged release of 95% and 48% within 28 h [77].

### 5.4. Complexation

**Figure 5:**
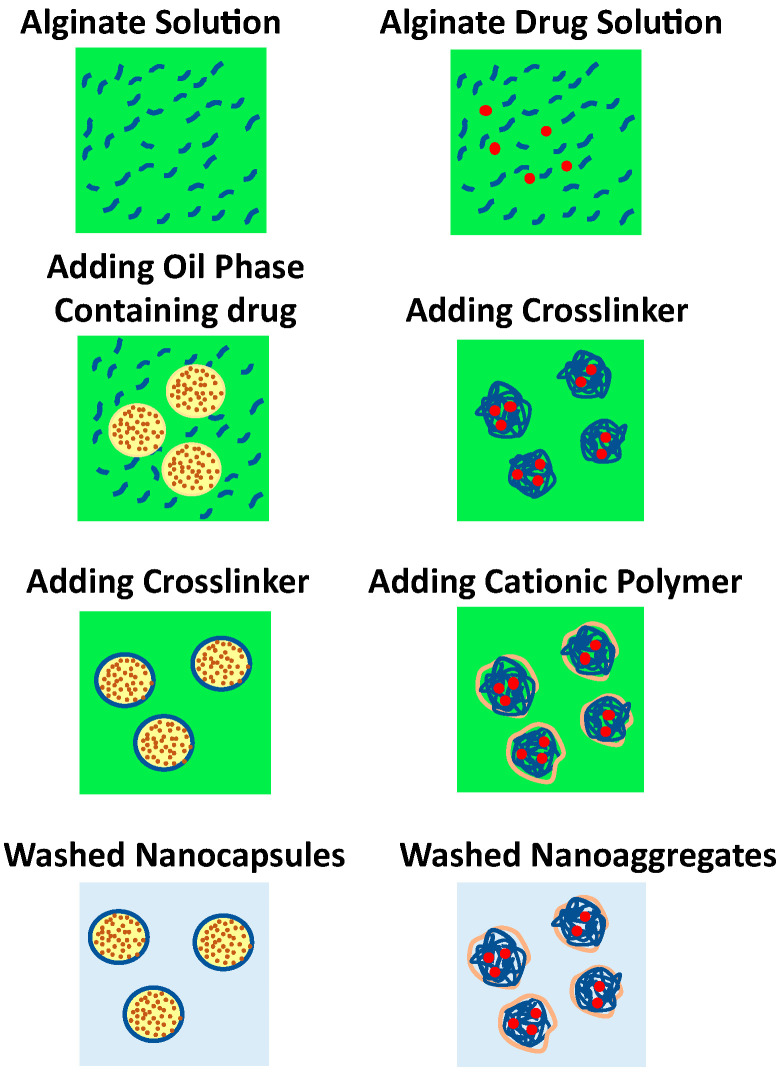
Schematic presentation of alginate nanocapsules and nanoaggregates formation via complexation method.

The complexation method produces two types of nanoparticles with reference to the medium used, where alginate nanoaggregates and nanocapsules are synthesized in an aqueous solution and on the interface of oil droplet, respectively. In general, complexation of alginate takes place using divalent ions like Ca^2+^ (available in calcium chloride), which act as a crosslinker and/or by incorporating of oppositely charged polyelectrolytes such as chitosan [78] and polymethacrylate [79]. The production of alginate nanoaggregates is attained by pre-gelation state, in which alginate solution is mixed with calcium chloride in aqueous continuous phase. In addition, complexation of alginate is also feasible by polyelectrolyte complex through mixing alginate and polycationic polymer such as chitosan and polymethacrylate to obtain polyelectrolyte complex coated-alginate nanoparticles (Figure 5) [80,81].

Commonly, nanocapsule is synthesized through forming a shell on template droplets by polymer deposition on their interface with subsequent solvent removal. The crosslinker is added to stabilize the polymer shell by physical or covalent intermolecular forces (Figure 5). Briefly, this method consists of an organic solvent mixed with a drug to be encapsulated to form the interior phase of nanocapsule. This mixture is slowly added to an alginate solution that contains surfactant such as Tween 80, where oil-in water (o/w) emulsion is prepared by means of sonication [82]. In addition, chitosan can be synergistically included together with the crosslinker to promote the encapsulation efficiency and reduce the porosity of the alginate nanocapsules [83]. Table 1 summarizes recent studies of alginate produced by means of complexation method.

### 5.5. Alginate as a Coating Material for Nanocarrier (Layer-by-Layer Approach)

Layer-by-layer is a bottom-up coating technique, in which the film is formed through construction layers of micro/nanometric thicknesses to produce core-shell system. This method involves electrostatic interaction between oppositely charged polyelectrolytes, where alternating adsorption of multi-layers cationic and anionic polymers occurs on flat substrates. In addition, this technique enables drug to be embedded between the layers, and hence promoting encapsulation efficiency and drug release control propensity with the reference of the physiochemical properties of the polymeric carrier used [92]. Layer-by-layer process can be influenced by various factors, such as pH of the medium, saturation adsorption time, polyelectrolyte concentration, adsorption temperature and salt concentration of polyelectrolyte solutions as well as the type of the matrix and core used [93].

In the study of Liu et al., nanoliposomes were coated with alginate and chitosan via layer-by-layer method to improve liposomal stability and prevent leakage of the payload [94]. In order to form the first layer, anionic nanoliposomes were added into chitosan solution (0.6% *w*/*v*) and then incubated for 1 h under gentle stirring. The subsequent layer was deposited by dropping chitosan-nanoliposomes into alginate solution (0.5% *w*/*v*) using the same procedure. The size, polydispersity index and zeta potential of the prepared nanoliposomes were around 89 nm, 0.26 and −6.3 mV, respectively. On the other hand, the final formulation of coated nanoliposomes exhibited larger mean size at 330 nm, polydispersity index at 0.37 and zeta potential at −15.8 mV in pH 5.5. The enzymatic digestion stability test demonstrated that coated nanoliposomes have conferred lipolytic degradation resistance and delayed release of encapsulant in simulated gastrointestinal conditions compared to coat-free nanoliposomes. 

In order to control doxorubicin release for antitumor activity, Chai et al., have prepared alternative multilayer of chitosan (cationic polymer) and alginate (anionic polymer) on the surface of doxorubicin-loaded PLGA nanoparticles. The influences of temperature, polyelectrolyte polymers concentration and NaCl concentration on the multilayer growth were investigated. Doxorubicin PLGA nanoparticles were prepared via double water in-oil-in-water (w/o/w) emulsion-solvent evaporation method. Subsequently, chitosan and alginate were alternately deposited on the nanoparticle surface to form multilayers of polyelectrolyte. It was found that, increasing polyelectrolyte or NaCl concentration as well as the adsorption temperature, the coat weight of the multilayer film was intensified. The initial burst release was significantly reduced as a result of employing layer-by-layer technique, while lowering the pH of dissolution medium conferred an increase of doxorubicin release [95].

Wang et al., have modified paclitaxel-PLGA nanoparticles prepared via solvent evaporation method by depositing chitosan and alginate layer-by-layer to improve the drug burst release tendency. After chloroform removal (volatile solvent) from w/o single emulsion, chitosan solution (0.5% *w*/*v*) was added dropwise into the emulsion under continues stirring to adsorb the first coat layer on the surface of nanoparticles by electrostatic force. In order to form a second coat layer, alginate solution (5 mg/mL) was added to PLGA-chitosan nanoparticles solution and ultrasound sonicated for 10 min. Three types of nanoparticles (PLGA, PLGA/chitosan and PLGA/chitosan/alginate) were separately prepared and characterized with regard to mean size, zeta potential, encapsulation efficiency and loading capacity. The particle size, encapsulation efficiency and loading capacity were increased proportionally after depositing each layer. The values of zeta potential of the above-mentioned nanoparticles were −2.72 ± 0.17 mV, +17.36 ± 0.84 mV and −10.62 ± 0.38 mV, respectively. The drug release study was carried out in PBS buffer (pH 7.4), and paclitaxel nanoparticles exhibited low initial burst release and prolonged release properties [44]. Khan et al., have investigated the influence of alginate/chitosan coat complex on the sustained release and bio-accessibility of resveratrol-zein nanoparticles [96]. The average particle size and zeta potential of initial nanoparticles were increased from 16.9 to ~72 nm and from +15.01 to +43.01 mV, respectively, while the effect on the encapsulation efficiency was negated. Besides, the release of resveratrol was sustained under simulated gastrointestinal condition, and its bio-accessibility was improved significantly.

### 5.6. Spray Drying

**Figure 6:**
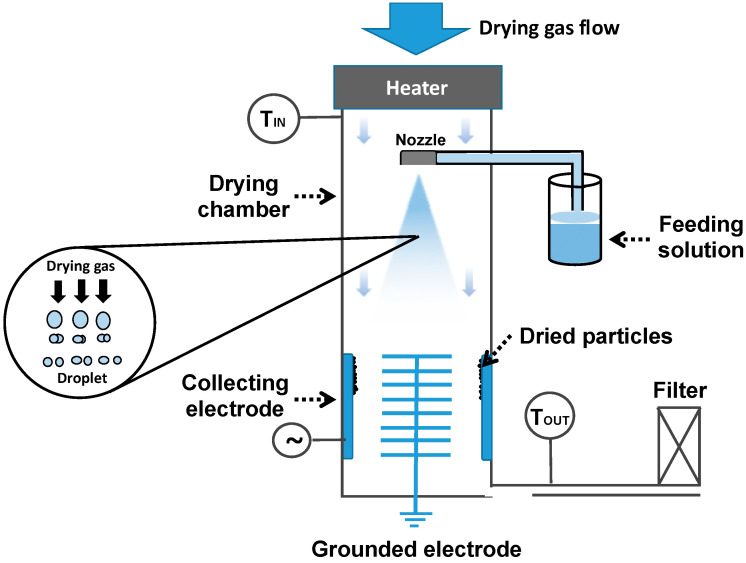
Schematic presentation of the conventional spray-drying technique.

Spray drying is one of the common techniques of micro and nanoparticle production where a liquid is atomized to droplets and dried using a hot gas [97]. In 1872, Samuel Percy was the first person to patent a spray drying technique that has been developed concerning its safety and productivity [98]. The production of nanoparticles using spray drying process is based on the removal of moisture from sprayed wet droplets by using a heated atmosphere. The working principle of a spray dryer includes four basic steps: emulsifying, dissolving or dispersing the drug in solvent (i), atomization of the solution into a spray using specific nozzle (ii), drying the sprayed droplets by drying gas (iii), and collecting the product (iv) [99]. The drying gas is introduced via an air dispenser from top of the chamber. At a constant flow rate and appropriate temperature, the feed solution is atomized into the drying gas chamber, in which the wet fine droplets are dried by moisture vaporization. Dry particles are collected through electrostatic particle collector that confers the particle surface charged and deflects by an electric filed. The collector consists of a rounded stainless still tube linked to a high voltage supplier (anode) and a grounded star electrode (cathode) inside the tube. Finally, the exhausted gas is passed through outlet filter that traps free particles from the gas (Figure 6) [100]. 

Alfatama et al., prepared three types of nanoparticles for oral delivery of insulin via spray drying technique. Simple alginate, alginate-C18 and alginate-stearic acid were used to obtain spherical nanoparticles with particle size of 513 nm, 522 nm, 619 nm, insulin encapsulation efficiency of 44.4%, 44.9%, 76.7% and polydispersity index of 0.54, 0.74, 0.33, respectively. The processing parameters used were as follows: air flow rate 2–2.5 m/s, solution feed rate 4 mL/min and atomizing air pressure 6 bar. The inlet temperature and outlet temperature were 60 °C and 23 °C, respectively [57]. Shehata and Ibrahima, on the other hand, have encapsulated metformin hydrochloride into nanoparticles consisting of alginate/gelatin with 1:3 ratio using nano-spray dryer. The prepared nanoparticles exhibited a mean diameter around 850 nm, polydispersity index 0.14, yield 81% and encapsulation efficiency 90%, employing nano-spray parameters: spray cap 7 µm, flow rate 7 mL/min, flow of drying gas 110 L/min with relative flow rate 100%, inlet drying gas temperature 120 °C, outlet temperature 35 °C, and actuator 60 kHz to form droplets through vibration membrane. The obtained dried nanoparticles manifested good flowability with an angle of repose around 31° due to their spherical shaped, however the reduced yield was attributed to the nanoparticles powder that stuck to the scraper and the electrode in the collection chamber [41].

In another study by A. El-Missiry et al., ellagic acid nanoparticles were synthesized using calcium-alginate via nano-spray drying and followed by ionotropic gelation with calcium ions. Alginate and ellagic acid were used at mass ratio 1:1 with alginate concentration at 0.025% *w*/*v*. The spray drying condition used: inlet temperature 120 °C and an air flow rate 135 L/min. The obtained nanoparticles were weighted and added into calcium chloride solution (0.1% *w*/*v*) as a crosslinker. The results showed that the nanoparticles mean size was around 670 nm [101].

De Cicco et al., have encapsulated gentamicin sulfate into alginate pectin nanoparticles via nano-spray dryer technique after adjusting feed rate and nozzle spray mesh size. The process conditions and parameters used: inlet temperature 90 °C, air flow 100 L/min, feed rate 9.5 mL/min, relative spray rate 100% and nozzle size 4.0 µm, 5.5 µm or 7.0 µm. Modulating nozzle size has directly impacted the particles size and particles size distribution proportionally, while the feed rate has affected the particles size distribution only in symmetrical manner. Apparently, the above-mentioned parameters had no significant effect on the encapsulation efficiency [102]. In the pharmaceutical industry, spray drying technique plays a significant role in manufacturing of drug powders and other therapeutic products, and this technique, in particular, can produce a high drug encapsulation efficiency and operate under sterile conditions [103,104]. It has also become an attractive technique in other industries such as cosmetics [105], food [106], and photoluminescence [107].

### 5.7. Electrospray

**Figure 7:**
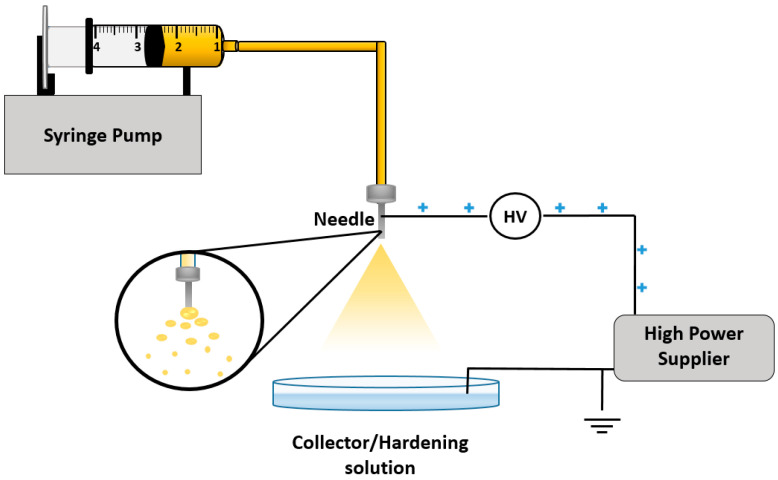
Schematic representation of electrospray technique.

Electrospray technique exhibits unique advantages to develop micro- and nanoparticles due to the friendly single step approach, the ability to control the particle size, the low amount of solvents and the yield control [108]. This technique is booming in the research and industry fields because of its ability to produce monodisperse droplets from nano-size to hundreds of micrometers, depending on the processing parameters [109,110]. Several researchers have successfully employed this technique to encapsulate macromolecular bioactive agents such as cells [111], proteins [112], nucleic acids [46] and others. Firstly, a liquid is pumped slowly through a thin metal needle using a syringe pump by a constant flow rate. Then, high voltage is applied on the needle to increase the acceleration of exited liquid away from the needle and overcome the surface tension of droplets. Finally, the droplets at the tip of the needle are converted into fine nano/micro-sized spray and form a cone called the Taylor cone (Figure 7) [113]. The size of the final droplet can be controlled by modulating the processing parameters, such as the flow rate, voltage, needle size, and the distance between the needle and the surface of the collector, and formulation parameters, such as materials concentration, crosslinkers, and surfactants. This method offers many advantages, such as using small input materials to get high yield of nanoparticles and thus avoiding waste of expensive substances, a single continuous one-step approach, and low cost, while the disadvantage is associated with time consuming [114].

Tsai and Ting have synthesized alginate and alginate/chitosan bilayer nanoparticles via electrospray technique. Central Composite Design-Response Surface Methodology (CCD-RSM) was applied to investigate the influence of flow rate, applied voltage, needle distance and alginate concentration on the particle diameters. The results reported that the size of alginate nanoparticles was significantly influenced by the applied voltage and the concentration of alginate solution, whereas the effects of the distance between the needle to the surface of the receiving medium and flow rate were negated. The optimum parameters produced alginate nanoparticles with size 279.17 ± 16.33 nm and zeta potential −57.3 ± 0.15 mV via modulating the flow rate, collector distance, applied voltage and alginate concentration are 0.6 mL/h, 20 cm, 27 kV and 3% *w*/*v*, respectively. Finally, chitosan/alginate bilayer nanocarrier were synthesized using chitosan as a core and alginate as a shell. Electrospray nozzle comprising two concentric stainless-steel needles (coaxial needle) was utilized to produce nanocarrier with dual layer, in which, the outer needle diameter 0.96 mm (for shell), while the inner needle diameter 0.52 mm (for core). The processing parameters were as follows: concentration of chitosan and alginate solutions at 2% *w*/*w* and 3% *w*/*w*, respectively, the distance of collector 20 cm, and voltage 27 kV. Taylor cone structure consisted of two solutions was determined experimentally to be at ratio 1:11 of chitosan solution (core) and alginate solution (shell). The final results showed that chitosan/alginate nanocarrier were produced by electrospray technique with mean size particles 112.1 ± 35.2 nm, polydispersity 0.42 ± 0.06 and zeta potential +21.82 ± 2.23 mV [115].

In another study, B. Alallam et al., have formulated alginate nanoparticles as a carrier for plasmid DNA by means of electrospray technique [46]. The study reported that the particle size of the nanoparticles reduced significantly from 1500 nm to 420 nm as a function of voltage rise from 9 kV to 12 kV. This can be advocated that higher voltage brings about stronger coulombic forces which intensify repulsion between adjacent droplets, producing smaller droplets size. Furthermore, the effect of flow rate of atomized solution was also investigated and reported a decrease in particle size from 533 nm to 463 nm upon acceleration the flow rate from 0.1 mL/h to 0.5 mL/h. In order to find the optimum ratio between flow rate and applied voltage, the interaction effect was examined using Minitab software. The suggested parameters from the design were the highest voltage (12.5 kV) with the lowest flow rate of 0.1 mL/h. This can be explained by the uneven spread of the emulsion at the tip of the needle, caused by high flow rate, resulted in uncontrolled atomization and formation of large particles [116].

### 5.8. Electrospinning

**Figure 8:**
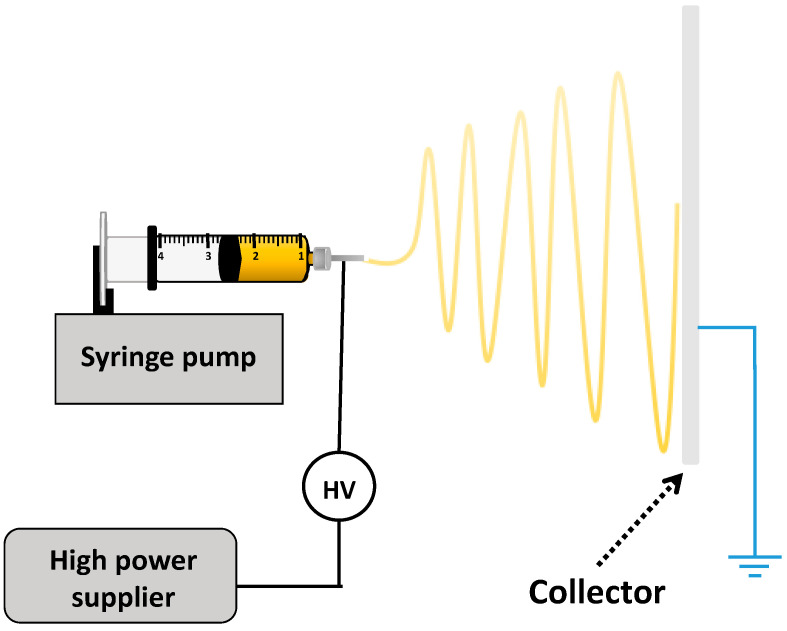
Schematic representation of electrospinning technique.

Electrospinning is a simple technique that produces nanofibers via electric fields to extract ultrafine fiber from polymer solution or melt [117]. This technique has attracted high attention in regenerative medicine, tissue engineering and industrial fields due to the ease of controlling the formed fiber properties such as the shape, size, and porosity. In addition, the nanofiber has been also applied in drug delivery system such as protein, antibiotics, DNA, RNA, living cells and growth factors, because it offers a high surface-to-volume ratio and ability to control drug loading and release profiles [118,119]. Electrospinning setup normally comprises of a syringe pump, high voltage power supply and collector that separated at a defined distance. In general, electrospinning process is considered as a sister technology with electrospray technique (Figure 8) [120]. The major difference between both techniques is the polymer concentration, in which high concentration can be used in electrospinning to obtain a more stable jet as well as elongation takes place by whipping instability mechanism [121]. The essential parameters that affect diameter of the electrospun and morphology of nanofibers are: needle diameter, distance between needle and collector, flow rate, solvent volatility and applied voltage [122]. Table 2 represents examples of previous research studies of alginate nanofibers synthesized via electrospinning method. 

## 6. Limitations of Alginate Nanofabrication

Despite the rapid progression and verities of techniques to synthesize alginate nanoparticles, it is still less striking compared to synthetic polymers due to extensive molecular weight variations and source-related properties [128,129]. Alginate purity determines the biocompatibility of the prepared nanoparticles, thus the presence of hazardous contaminants such as endotoxins, polyphenolic compounds, proteins and heavy metals, may cause an immune response in the site of administration [130]. In the market, alginates are available in different grades, purities, and qualities, based on the manufacturer and source [131]. Moreover, the difference in M/G blocks ratios of alginate has been correlated to innate immune system stimulation as a result of the presence of cytokines in response to high M block (β-d-mannuronic acid) content [132]. 

On the other hand, many studies have reported a poor encapsulation efficiency and high burst drug release of alginate nanoparticles [86,90,133]. This can be attributed to the hydrophilicity and high porosity of alginate nanoparticles leading to instability and swelling at biological fluids as well as leakage of entrapped drugs during the preparation process [18,134]. Different chemical modification approaches have been implemented to overcome these limitations, improving the mechanical strength of alginate nanoparticles and to confer a hydrophobic character of alginate [135,136]. However, this usually involves chemical reagents such as aldehydes, which may require purification prior to clinical uses [137]. Moreover, the use of organic solvents for alginate derivation may influence the physiochemical and biological characteristics of the encapsulant [138]. In addition, some of the preparation methods of alginate nanoparticles including emulsification-solvent displacement and solvent evaporation are mediated by organic solvents [128].

## 7. Factors Influencing Alginate Nanoparticles’ Characteristics: Particle Size, Size Distribution, Encapsulation Efficiency and Drug Release

### 7.1. The Influence of Alginate Concentration

Yasmin et al., have highlighted the effect of alginate concentration on the size, size distribution and encapsulation efficiency of nanospheres prepared by means of emulsification/external gelation method. The results revealed that alginate concentration has a significant effect on the mean diameter of nanospheres, in which alginate concentration 3% *w*/*v* and 5% *w*/*v* resulted in nanospheres with size range 700 nm to 900 nm, respectively. Further, high alginate concentration 5% *w*/*v* was associated with improved encapsulation efficiency of bovine serum albumin loaded-nanospheres. The results also showed that high alginate concentration is effective in reducing the initial burst release from 86 ± 3.62% to 74 ± 1.53%, while the effect of alginate concentration on the total protein release was negated [139]. Similar results were reported by Sarei et al., whereby direct proportional relationship between alginate concentration and size, encapsulation efficiency and polydispersity index of the prepared nanospheres was found, while a slow drug release was attainable. This phenomenon was observed and explained extensively, advocating the use of high alginate concentration produces high solution viscosity and low shear stress that lead to larger emulsion droplets [140]. In another study, Mokhtari et al., have analyzed the influence of alginate concentration on nanoparticle’s attributes prepared by emulsification/internal gelation technique. They have reported an increase in particle size from 512 nm to 4303 nm and encapsulation efficiency from 5.26% to 7.62%, when alginate concentration was raised from 0.5% *w*/*v* to 1% *w*/*v*, respectively [141]. This may be explicated by higher alginate concentration induced more forces in alginate droplets that may resist droplets breakdown and hinder the diffusion of drug from alginate droplets to the oil phase during preparation process [142]. Besides, nanocarriers, being relatively large in size and hydrophilic in nature, possess small specific surface area and are less prone to burst or premature release [143].

Novitasari et al., have synthesized alginate-chitosan nanoparticles by means of ionic gelation technique using calcium ions. The influence of the processing parameters was assessed using full factorial design to predict the effect of each factor and their interactions on the responses. Based on the results, high alginate concentration led to an increase in particles size and polydispersity index and improved encapsulation efficiency of the nanoparticles. With reference to the interaction effect among all factors (alginate, calcium chloride and chitosan concentration), the lowest particles size (200 nm) and polydispersity index (0.27) with encapsulation efficiency around 35% were attainable by reducing both alginate and chitosan concentrations while maintaining high value of calcium chloride concentration [144]. Similar findings were also reported by Zimet et al., when Nisaplin^®^ loaded alginate nanoparticles have been prepared by means of ionic gelation/complexation method [145]. The results showed an increase in particle size from 86 nm to 204 nm, zeta potential from −33.2 mV to −38.7 mV and a decrease in encapsulation efficiency from 35.6% to 30.5% when alginate concentration was increased from 0.03% *w*/*v* to 0.07% *w*/*v*, respectively. Encapsulation efficiency was directly proportional to Nisaplin^®^: alginate mass ratio of the nanoparticles.

Mansourpour et al., have applied factorial models to examine the influence of alginate concentration on the size and polydispersity index of nanoparticles produced by ionotropic gelation. Higher alginate concentration resulted in particles with larger size and higher polydispersity index, in which the effect of alginate concentration was driven by other independent factors namely, CaCl_2_ and cationic β-cyclodextrin concentrations [146]. Furthermore, Rahaiee et al., have investigated the effects of alginate and chitosan concentrations to optimize the characteristics of the prepared nanoparticle via ionic gelation method. The influence of alginate concentration on the size and encapsulation efficiency of the nanoparticles was dominated compared to other factors. Simultaneous rise of alginate and chitosan concentration rendered encapsulation efficiency to drop, but this was reversible by mere reducing chitosan concentration. This can be attributed to the competition of the drug and chitosan on the binding sites onto alginate moieties. On the other hand, the nanoparticle size was reducible by decreasing the concentration of alginate [133]. Another study by Govindaraju et al., indicated the role of alginate on curcumin loaded alginate nanoparticles synthesized by ionotropic gelation method [147]. They have reported that high alginate concentration was associated with a significantly increased zeta potential from +150 ± 1.15 mV to +200 ± 2.15 mV and a slightly changed size and encapsulation efficiency of the prepared nanoparticle. The positive value of zeta potential was elucidated by its relationship with the absorbed amount of Ca^2+^ after alginate neutralization by CaCl_2_.

Based on the abovementioned studies, it can be concluded that alginate concentration has the main influence on the size, polydispersity index and encapsulation efficiency of nanoparticles prepared via ionic gelation method. This influence can be advocated by the extent of interaction between the functional groups of alginate chains (COO^−^) and calcium ions. Higher alginate concentration produces particles with larger size and wider polydispersity index as a result of increasing the number of carboxylate groups and alginate chain layers which can intensify around calcium cations. This in turn creates additional space for more drug to be entrapped, and thus improves the encapsulation efficiency, as summarized in Table 3 [45,148].

### 7.2. The Influence of Surfactant

Mokhtari et al., highlighted the effect of variant concentrations of Tween 80 as a surfactant with hydrophile-lipophile balance (HLB) of 4.3 used to aid the encapsulation of peppermint phenolic extract in alginate nanospheres via emulsification/internal gelation method. The results indicated that higher surfactant content produced nanospheres with lower particles size and higher encapsulation efficiency as a result of decreasing the surface tension to promote breakdown extent of alginate droplets [141]. Another study by Elgegren et al., indicated the role of surfactant on sacha inchi (*Plukenetia volubilis* L.) oil-loaded alginate nanoparticles synthesized by emulsification/external gelation with complexation process. They have reported that high Poloxamer 407 concentration was associated with decreased both size and polydispersity index of the prepared nanoparticles [149]. Moreover, incorporation of Polysorbate 80 (10% *v*/*v*, 20% *v*/*v*, and 40% *v*/*v*) has rendered a rise in particle size, while the encapsulation efficiency peaked at medium concentration of the surfactant [147]. Similar results were also reported by Scolari et al., in which rifampicin-ascorbic acid-loaded alginate nanoparticles coated with chitosan were fabricated by ionic gelation/complexation method. The results reported a significant increase in particle size when the Tween 80 concentration increased from 0.25% *w*/*v* to 0.5% *w*/*v* [150].

Within a single assembled system, Baghbani et al., have investigated the effect of surfactant (Tween 20) and co-surfactant (Span 60/Poloxamer 188) on the nanoparticles attributes prepared by emulsification/external gelation method. Mere use of Tween 20 at low concentration (0.1% *v*/*v*) was undesirable as it did not inhibit the separation of the emulsion phases, while at higher surfactant concentration (0.3% *v*/*v*), smaller particle size and polydispersity index, improved encapsulation efficiency and delayed manner of drug release were achieved. Besides, the effect of co-surfactant inclusion (Span 60 or Poloxamer 188) at three different concentrations (0%, 0.15% *w*/*v* and 0.3% *w*/*v*) coupled with 0.3% *v*/*v* Tween 20 on the particle size, entrapment efficiency, and drug release kinetics of doxorubicin-loaded alginate nanodroplets was also investigated. The particle size was decreased and then increased upon using higher strength of Span 60 or Poloxamer 188 in the formulation from 0% to 0.15% *w*/*v*. Using high Span 60 concentration impacted the encapsulation efficiency and cumulative drug release negatively at a fixed concentration of Tween 20, while the effect was negated upon varying the amounts of Poloxamer 188. This can be explained by the hydrophobic nature of Poloxamer 188 that covers the oil nanodroplets more efficiently and reduces drug release by retarding the degradation of alginate through neutralizing the acidity produced as a result of its degradation [148].

The large size and low encapsulation efficiency of nanoparticles associated with low concentration of surfactant can be explained by the production of unstable droplets and high propensity of coalescence, as a results of irregular surface tension due to incomplete covering of the particle surfaces. On the other hand, high levels of surfactant lead to similar effects as above in addition to higher polydispersity index. This can be advocated by higher interaction tendency between polymers or hydrophilic chains of different particles [150,151]. In addition, reduced encapsulation efficiency upon increasing the amount of surfactant was also observed, it may be attributed to the reduction in the spaces within polymeric chains (as a result of generating reverse micelles inside it) as well as losing the entrapped drug during preparation as a function of low surface tension of droplets. Table 4 represents recent examples of the effect of surfactant on the nanoparticle’s attributes [141].

### 7.3. The Influence of CaCl_2_ Concentration

Mokhtari et al., evaluated the effect of CaCl_2_ concentration on the size and encapsulation efficiency of peppermint phenolic extract-loaded nanospheres prepared by means of emulsification/internal gelation. The results reported a decrease in the mean particle size and enhancement of encapsulation efficiency as a function of increasing the molarity of CaCl_2_ [141]. For the development of metronidazole loaded chitosan-alginate nanoparticles, Sabbagh et al., applied a full factorial design to achieve the optimum concentration of CaCl_2_ and other factors related to ionotropic pre-gelation process. The study reported that high concentration of CaCl_2_ led to formation of smaller particle size and improved loading efficiency as well as reduced zeta potential [152]. Similar findings were also observed by Ahdyani et al., in which timolol maleate-loaded chitosan-alginate nanoparticles were prepared via ionic gelation technique. The results indicated reduction in particle size when CaCl_2_ concentration was increased from 0.05% *w*/*v* to 0.25% *w*/*v* (Table 5) [144].

The crosslinking process has a significant impact on the physiochemical properties of alginate hydrogel, in which the carboxylic groups of alginate backbone interact preferentially with calcium ions to form a stable three-dimensional network [146,153]. The higher cross-linking concentration induces shorter polymer chain and more network density of gel matrix, resulting in smaller size and size distribution as well as improved drug release extent of alginate particles [154]. Besides, the decrease in calcium cationic ions improves zeta potential of particle surface to be more negative, conferring better stability, drug loss retardation, and high encapsulation efficiency [80,155].

### 7.4. The Influence of Crosslinking Time

The effect of varying crosslinking times on the properties of bovine serum albumin-loaded alginate nanospheres, prepared via emulsification/external gelation method was assessed [139]. The results demonstrated that prolonging gelation time from 1 min to 10 min led to a slight reduction of the particle size and initial burst release profiles, while encapsulation efficiency was improved (43 ± 7.50% to 51 ± 8.09%), however, further lengthening of the processing time exhibited a counteractive effect. This can be attributed to the completion of alginate gelation within 10 min, whereas a shorter stirring time was insufficient [156,157]. On the other hand, longer crosslinking time resulted in diffusing more Ca^2+^ ions into alginate nanospheres that led to an increase in viscosity of the alginate phase, increased porosity, more junction zones and, hence a leakage of drug molecules from the alginate droplets to the medium [158,159].The effect of the complexation time was examined at 90 min, 120 min, and 180 min with respect to the particle size and entrapment efficiency to produce streptomycin-loaded alginate-chitosan nanoparticles via ionotropic complexation method. It was found that the lowest particles size (374 nm) and the highest encapsulation efficiency (93.32%) were achieved at complexation duration of 90 min [160]. However, the effect of complexation time on the encapsulation efficiency of trans-cinnamaldehyde nanoparticles prepared by combination of ionic gelation and complexation techniques, according to Loquercio study, was negated, while the mean particle size (294.78 nm) was reducible by increasing the crosslinking duration from 45 min to 90 min [20].

Another study was conducted to evaluate the particle size of insulin nanoparticles synthesized via emulsification/internal gelation. Similar trend of results was observed where the reduction of particle size (442 nm to 317 nm) was enabled by applying ultrasonication for 15 min, after which the size started to expand [161]. To enable a marked reduction of particle size, it was envisaged that ultrasound sonication brought about emulsion droplet breakdown by means of cavitation phenomena, yet lengthening this process time rendered the coalescence of emulsion droplets as a result of elevating the medium temperature and hence larger particle size [162].

The short stirring duration may not be adequate to generate intensive electrolyte interactions that compact the polymer chains on the nanoparticles surface [163]. This may lead to the production of larger particles with exposed pores that could result in the loss of the encapsulant during preparation [18,164]. On the other hand, longer stirring time might cause aggregation of polymer molecules on the surface of nanoparticles and reduction of free spaces within alginate matrix [157].

### 7.5. The Influence of Stirring Rate

J. Emami et al., have evaluated the effect of stirring rate from 500 rpm to 2000 rpm on the mean particle size, encapsulation efficiency and drug release profiles of glipizide-loaded alginate-chitosan nanoparticles using ionotropic controlled gelation technique. The results showed a negative influence on average particles size and encapsulation efficiency of alginate nanoparticles, while drug release was remarkably prolonged by increasing the stirring rate [165]. Similar findings were reported by other researchers in which the nanoparticle diameter decreased from 627 nm to 236 nm as a function of intensifying stirring rate from 500 rpm to 1000 rpm [166].

In another study, Samprasit et al., have prepared mangostin-loaded chitosan-alginate nanoparticles via ionotropic gelation method. It was found that an increase of stirring speed from 1000 rpm to 1400 rpm has resulted in a significant decrease in nanoparticle mean size [167]. Mohamed and Laraba-Djebari have also prepared calcium alginate nanoparticles as vaccine delivery by means of ionic gelation method. The concentration of alginate, CaCl_2_ and the time of homogenization remained constant, while the homogenization rate varied from 500 to 1500 rpm. The results indicated that higher homogenization extent conferred a significant decrease in the size of the nanoparticles from <1000 to 85–300 nm [168].

From the previous studies, it can be concluded that stirring speed impacted the particle size significantly. This phenomenon can be evidently observed by transfer of mechanical energy of different stirring rates, in which the breaking energy is increased upon rising the stirring speed, resulting in smaller particles size [169,170]. The variability in the drug release profiles can be indicated by the variance in the particle size [171,172]. Smaller particles have a faster drug release tendency due to their large surface-to-volume area, as well as shorter diffusion pathways that transfer the payload to the outer dissolution medium [148].

### 7.6. The Influence of pH

In general, the complex of anionic and cationic ions occurs based on the electrostatic interactions between charged molecules [173]. The strength of the polyelectrolyte complex is influenced by the pH value of the solution [174]. In order to find the optimum pH value that offers the highest number of ionized or protonated groups required for interaction, the pH range should be investigated [175]. The pKa value of alginate is between 3.4 and 4.4 (depending on its source). At pH > pKa, the carboxylate groups of alginates are ionized (Alg-COO^−^) and electrostatically linked to calcium ions and cationic polymers [92]. Furthermore, the negatively charged carboxylate groups are predominant at weak acid medium around pH = 5 [85]. While, amino groups (NH_2_) of chitosan are protonated (NH_3_^+^) at pH below its pKa (6.5), it allows chitosan to coacervate with anionic polymers such as alginate [176]. As shown in Table 6, alginate-chitosan nanoparticles with small size and high encapsulation efficiency were produced at halfway between pKa of these species (pH ~ 4.8). Besides, intensifying of electrostatic interactions is represented by zeta potential values, in which high negative values indicate an absence of free cationic groups on the surface of nanoparticles, that correlated to high encapsulation efficiency and drug loading propensity [161].

### 7.7. The Influence of Alginate: Chitosan Mass Ratio

Table 7 represents recent examples of nanoparticles prepared from alginate/chitosan blends via different approaches. It can be evidently noticed that higher amounts of chitosan produce larger particles as well as lower encapsulation efficiency. This can be explained by the higher affinity of amino groups of chitosan to M residues than G residues of alginate [133,163,178]. Hence, reducing the alginate:chitosan ratio may lead to a competition between amino groups and cationic Ca^2+^ ions on the G block binding sites after saturation of the binding sites of M residues of alginate. This may bring about particles aggregation which could have a negative impact on the encapsulation efficiency and loading capacity, as well as an increase in particles size and zeta potential. On the other hand, decreasing the alginate:chitosan ratio can minimize the electrostatic attraction between them, suppresses encapsulation ability and drug release control as well as increase the particle size [133,179].

## 8. Comparison of Alginate Nanoparticles’ Synthesis Methods

Hydrophobic and hydrophilic molecules such as anti-cancer drugs (doxorubicin [89], crocin [86], curcumin, resveratrol [88], exemestane [87]) were successfully loaded into alginate nanoparticles via complexation method. Emulsification/gelation method on the other hand, is preferable to encapsulate hydrophobic substances due to the presence of two phases. This method enables synthesis of relatively small size nanoparticles as low as 39.2 nm [148]. In addition, nozzle-based methods such as spray drying [106], electrospray [182] and electrospinning [183] are able to produce nanoparticles of reduced polydispersity index and improved encapsulation efficiency due to the fact of single-step method where drug loss is minimized. Table 8 summarizes the characterization and formulation/processing factors that can be modulated to obtain an optimal formulation of alginate nanoparticles for specific drug delivery system.

## 9. Conclusions

Alginate possesses great biocompatibility and approved as food additive by the US-FDA, thus it is a preferred candidate among pharmaceutical excipients for designing of advanced drug delivery system for oral delivery. The high interest of the scientific community in alginate synchronized with the revolution in therapeutics-driven by nanomedicine. The present spotlight review highlighted the most recent studies of alginate as a platform to develop nanoparticles intended for oral administration. The most elegant attributes are mucoadhesive and mucopenetration that promote the passage of payloads through the gastrointestinal epithelium. This could be availed to improve local and systemic delivery, enhance oral bioavailability, and control drug release. Even the research of alginate nanoparticles is less profuse and has been limited to a few cargo substances compared to microparticles, and various techniques have been adopted to design different types of nanocarriers. The selection of the polymers and formulation technique mainly relies on the pharmaceutical excipients and application goal, such as improving payload delivery and enhancing sustained release and site-targeting. Generally, emulsification/gelation is a low-cost technique for producing small particles in large quantity, involving preparation of alginate-in-oil (w/o), followed by gelation process. Polyelectrolyte complexation method on the other hand, is the simplest and most common technique to prepare alginate nanoparticles, where oppositely charged polyelectrolyte complex takes place. Moreover, layer-by-layer technique mainly used in drug targeting delivery and controlled drug release systems. In addition, nozzle-associated methods are based on extruding the polymer solution from the nozzle tip, including nano-spray dryer, electrospray and electrospinning. Careful selection of recipe and processing parameters is essential to formulate nanocarrier with tuned attributes. Additionally, future perspectives in polymeric nanoparticles should concentrate on studies of employing new and most performing techniques to develop advanced delivery systems, thereby expanding the applications of polymeric nanostructure in pharmaceutical field. To conclude, alginate endures a great potential and its extensive implementation in the advancement of innovative nanocarrier delivery systems with translation possibility is a matter of time.

## Figures and Tables

**Table 1:** Example of polymeric nanoparticles formulation by complexation method.

Alginate Concentration (% *w*/*v*)	Components	Drug	Aims	Mass Ratio	pH	Mean Particle Size (nm)	Polydispersity Index (%)	Zeta Potential (mV)	Encapsulation Efficiency (%)	Drug Release	Reference
-	CaCl_2_% *w*/*v*	Doxorubicin	Site-targeting and controlled release	-	-			-		-	[84]
0.04	0.39					350	0.481		65.0		
0.05	0.39					479	0.139		68.0		
0.06	0.39					490	0.273		71.0		
0.08	0.19					3997	1		77.0		
0.10	0.19					6638	1		84.0		
0.30	CaCl_2_ 0.10% *w*/*v* Span 80 Iron (1%)	Ferrous sulphate	To protect ferrous from oxidation oral iron therapy	-	~5.0	20 ± 6	-	−38.0 ± 4	95.0 ± 4	20% at pH 2.0 for 100 h 65% at pH 6.0 for 100 h 70% at pH 7.4 for 100 h	[85]
0.10	Chitosan (Cs) 0.08% *w*/*v*	Crocin	To improve bioavailability, anticancer, and antioxidant activity	-	4.70	236	0.476	-	38.16	30% at pH 1.2 for 48 h 50% at 6.8 for 48 h	[86]
0.50	CaCl_2_ 2% *w*/*v*	Exemestane	To reduce and control the release of exemestane	-	-	197	-	−18.3	98.0	Maximum release within 7 h at pH 7.4	[87]
0.06	CaCl_2_ 0.05% *w*/*v* Tween 80	Curcumin and resveratrol	Site-targeting	-	-	12.53 ± 1.06	-	−22.0 ± 2.17	49.30 ± 4.3	Curcumin 16.35 ± 3.8% for 24 h	[88]
						60.23 ± 15			70.99 ± 6.1	Resveratrol 87 ± 7% for 24 h	
0.30	Chitosan 0.08% *w*/*v* CaCl_2_	Doxorubicin	Site-targeting and controlled release	Alg:Cs 10:1	Alginate (Alg) 5.30	~300	0.2	−22.5 to −25.0	~97	52% at pH 5.5 for 6 h 35% at pH 7.4 for 6 h	[89]
					Cs 4.50						
0.1	CaCl_2_ 0.1% *w*/*v* Chitosan 0.1% *w*/*v*	ε-polylysine (ε-PL)	Evaluating the possibility of Cs/Alg nanoparticles as carriers of ε-polylysine	Alg:Cs 4.93:1	Alg 5.14	Alg-Cs 276.38	0.24	−33.7	53.37	17.5% at pH 6.6 for 2 h 80% at pH 6.6 for 10 h 90% at pH6.6 for 25 h	[90]
				Alg:ε-PL 100:8.55	-	ε-PL-Alg-Cs 372.05	0.29	−30.3			
-	Polyurethane-alginate (PU:Alg) CaCl_2_ 0.5% *w*/*v* Chitosan 1% *w*/*v*	Insulin	Enhancing potential of oral insulin delivery	PU:Alg 7:3	5.10	90–110	-	+38.5	90.0	15% at pH 1.2 for 2 h 50% at pH 6.8 for 10 h 100% at pH 7.4 for 20 h	[91]

PDI: polydispersity index; EE: encapsulation efficiency; Alg: alginate; Cs: chitosan; ε-PL: ε-polylysine; PU: polyurethane; SGF: simulated gastric fluid; SIF: simulated intestinal fluid; Ref: reference.

**Table 2:** Preparation of alginate nanofiber by electrospinning technique.

Formulation	Voltage (kV)	Needle Size (Gauge)	Flow Rate (mL/h)	Distance (cm)	Fibers Diameter (nm)	Reference
Sodium alginate 3% *w*/*w* Polyethylene oxide 3% *w*/*w* Triton X-100 0.5% *w*/*w* Dimethylsulphoxide 5% *w*/*w*	25	-	0.7	18	97.4	[123]
Sodium alginate 2% *w*/*v* Polyvinyl alcohol 14% *w*/*v* Nano-hydroxyapatite	11	21	0.32	17	270	[124]
Sodium alginate 1.5% *w*/*w* Polyethylene oxide 1.5% *w*/*w*	18	23	2	6	288	[125]
Sodium alginate 1.5% *w*/*w* Polyethylene oxide 1.5% *w*/*w* Carboxyl multi walled carbon nanotubes					280	
Polyethylene oxide 3% *w*/*w* Sodium alginate 2% *w*/*w*	10.5	180 μm	0.25	14	300	[126]
Sodium alginate 1.74% *w*/*w* Triton X-100 1.1% *w*/*w* Polyethylene oxide 0.43% *w*/*w* Dimethyl sulfoxide 5.43% *w*/*w*	12	27	0.6	12	240	[47]
Sodium alginate 2% *w*/*v* olyvinyl alcohol 10% *w*/*w*	26	25	0.48 0.6	10	62 180	[127]

**Table 3:** The influence of alginate concentration on the properties of its nanoparticles.

Alginate Concentration (% *w*/*v*)	Preparation Method	Drug	Mean Particle Size (nm)	Polydispersity Index (%)	Zeta Potential (mV)	Encapsulation Efficiency (%)	Reference
3	Emulsification/external gelation	Protein	700	-	-	47	[139]
5			900			51	
0.50	Emulsification/internal gelation	Peppermint	512	-	-	Increase	[141]
1			4303				
0.05	Ionic gelation/complexation	Crocin	Increase	-	-	Increase	[133]
0.30							
0.1	Ionic gelation/complexation	Timolol Maleate	473.1	0.37 ± 0.05	-	33.71 ± 4.7	[144]
0.5			489.3	0.51 ± 0.1		39.01 ± 2.8	
0.03	Ionic gelation/complexation	Nisaplin^®^	86	-	−33.2	35.6	[145]
0.07			204		−38.7	30.5	
0.1	Emulsification/external gelation	Doxorubicin	39.2	0.19	-	92.2	[148]
0.2			149.6	0.38		98.4	
0.01	Ionotropic pre-gelation/complexation	Insulin	Increase	Increase	-	-	[146]
0.1							
0.6	Ionotropic gelation	Curcumin	105	-	150 ± 1.15	94 ± 4.2	[147]
0.8			107		200 ± 2.15	92 ± 3.6	
1.0	Electrospray	-	315.9 ± 37.5	0.24 ± 0.10	-	-	[115]
2.0			348.2 ± 63.9	0.28 ± 0.03			

PDI: polydispersity index; EE: encapsulation efficiency; Ref: references.

**Table 4:** The influence of surfactant type/concentration on alginate nanoparticles.

Surfactant	Surfactant Concentration	Method of Nanoparticle Preparation	Drug Loaded	Mean Particle Size (nm)	Polydispersity Index (%)	Encapsulation Efficiency (%)	Reference
Tween 20	0.20% *v*/*v*	Emulsification/external gelation	Doxorubicin	102.4	0.25	87.2	[148]
	0.30% *v*/*v*			39.2	0.19	92.2	
	0.40% *v*/*v*			93.5	0.26	85.4	
Span 60 co-surfactant	0% *w*/*v*			51.8	0.23	93.5	[148]
	0.15% *w*/*v*			42.3	0.26	84.6	
	0.30% *w*/*v*			95.1	0.24	76.2	
Poloxamer 188 co-surfactant	0% *w*/*v*			51.8	0.23	93.5	[148]
	0.15% *w*/*v*			35.6	0.29	92.1	
	0.30% *w*/*v*			48.4	0.30	90.8	
Poloxamer 407	0.10% *w*/*v*	Emulsification/external gelation complexation	Sacha inchi oil	900	-	Decrease 0.1 to 0.3% *w*/*v* Increase 0.5 to 1% *w*/*v*	[149]
	0.20% *w*/*v*			1050			
	0.30% *w*/*v*			1000			
	0.50% *w*/*v*			700			
	1% *w*/*v*			800			
Polysorbate 80	10% *v*/*v*	Ionotropic gelation	Curcumin	Increase	-	92	[147]
	20% *v*/*v*					94	
	40% *v*/*v*					91	
Tween 80	0% *w*/*v*	Ionic gelation	Rifampicin Ascorbic Acid	450	-	-	[150]
	0.20% *w*/*v*			250			
	0.40% *w*/*v*			700			

**Table 5:** The influence of CaCl_2_ concentration on alginate nanoparticles.

CaCl_2_ Concentration (% *w*/*v*)	Preparation Method	Drug	Particle Size (nm)	Polydispersity Index	Zeta Potential (mV)	Encapsulation Efficiency (%)	Reference
0.05	Emulsification/internal gelation	Vegetable Oils	361	-	-	5.10	[141]
0.15			140			6.66	
0.05	Ionic gelation/complexation	Timolol Maleate	473	0.37	-	33.71	[144]
0.25			200	0.27		35.23	
3.0	Ionotropic pre-gelation/complexation	Metronidazole	Decrease	-	Less negative	-	[152]
6.0							
0.5	Ionotropic gelation/complexation	Insulin	Increase	Increase	-	-	[146]
3.0							

**Table 6:** The influence of pH on the prepared alginate nanoparticles.

Drug	Preparation Method	Formulation Composition	pH	Particle Size (nm)	Encapsulation Efficiency (%)	Zeta Potential (mV)	Reference
Crocin	Ionic gelation	Alginate 0.025% *w*/*v* Chitosan 0.04% *w*/*v*	5.16 4.74	341 268	30.7 33.1	-	[133]
Insulin	Ionotropic pre-gelation	Alginate Chitosan Cationic β-cyclodextrin	4.90	150.82	93.2	-	[146]
Blank/ε-polylysine	Ionic gelation	Alginate 0.1% *w*/*v* Chitosan 0.1% *w*/*v*	5.14	276.38	54.18	−33.7	[90]
Blank/doxorubicin	Ionic gelation	Alginate 0.3% *w*/*v* Chitosan 0.075% *w*/*v*	5.30	352	90	−32	[89]
Curcum	Layer-by-layer	Chitosan layer 200 mL Alginate layer 150 mL	3.0 5.0 7.0	-	-	+1 −30 −27	[92]
Curcumin diethyl diglutarate	o/w Emulsification and ionotropic gelation	Alginate 0.6 mg/mL/pH = 4.9 Chitosan/pH = 4.6 Pluronic^®^F-127 (surfactant)	-	215	85	−24.1	[177]

**Table 7:** Influence of alginate:chitosan (*w*/*w*) mass ratio on alginate nanoparticle.

Drug	Preparation Method	Mass Ratio	Particle Size (nm)	Encapsulation Efficiency (%)	Zeta Potential (mV)	Reference
Nisin	Ionic gelation	8:2	40	15.9	−45.6	[145]
	complexation		472	15.1	−29.8	
Curcumin diethyl disuccinate	Emulsification ionotropic gelation	1:0.05	279 ± 71	38.7 ± 2.8	−27.8 ± 0.3	[180]
		1:0.15	434 ± 17	17.1 ± 2.3	−19.8 ± 1.4	
Insulin	Alginate/chitosan core shell nanoformation	3:1	-	63.0	-	[91]
		3:2		71.0		
		3:3		77.0		
lovastatin	Ionic gelation	6:3	900 ± 101	-	-	[181]
		6.5:3	86 ± 3.7			
		7:3	220 ± 17.5			
-	Electrospray	11:1	112 ± 35	0.42 ± 0.06	-	[115]
		7:1	259 ± 68	0.34 ± 0.12		

**Table 8:** Summary of characterization and formulation/processing factors that influence the formation and properties of alginate nanoparticles.

Preparation Method	Nanoparticles Size Range (nm)	Polydispersity Index	Encapsulation Efficiency (%)	Formulation and Processing Factors	Reference
Complexation	20 ± 6 nm to 372.05 nm	0.2 to 0.476	38.16% to 98.0%	Alginate concentration CaCl_2_ concentration pH value Alginate: chitosan mass ratio	[85,86,87,89,90]
Emulsification/gelation	39.2 nm to 407.67 ± 19.18 nm	0.204 to 0.42 ± 0.15	81.70 ± 6.64% to 92.2%	Alginate concentration Type/concentration of surfactant/cosurfactant CaCl_2_ concentration Stirring time	[69,70,77,148]
Spray dryer	350 nm to 670 nm	0.54 to 0.74	44.4% to 80%	Alginate concentration Air flow rate Solution feed rate Inlet temperature Outlet temperature Nozzle spray mesh size	[41,101,102]
Electrospray	112.1 nm to 228 nm	0.17 to 0.43	~99%	Alginate concentration CaCl_2_ concentration Nozzle size Flow rate Distance between needle tip and collector Applied voltage	[46,115]
Electrospinning	62 nm to 300 nm	-	-	Alginate concentration Nozzle size Flow rate Distance Applied voltage	[126,127]

## References

[1] Jawahar N., Meyyanathan S. (2012). Polymeric Nanoparticles for Drug Delivery and Targeting: A Comprehensive Review. Int. J. Health Allied Sci..

[2] El-Say K.M., El-Sawy H.S. (2017). Polymeric Nanoparticles: Promising Platform for Drug Delivery. Int. J. Pharm..

[3] Witzigmann D., Kulkarni J.A., Leung J., Chen S., Cullis P.R., van der Meel R. (2020). Lipid Nanoparticle Technology for Therapeutic Gene Regulation in the Liver. Adv. Drug Deliv. Rev..

[4] Poovi G., Damodharan N. (2018). Lipid Nanoparticles: A Challenging Approach for Oral Delivery of BCS Class-II Drugs. Future J. Pharm. Sci..

[5] Wu K., Su D., Liu J., Saha R., Wang J.P. (2019). Magnetic Nanoparticles in Nanomedicine: A Review of Recent Advances. Nanotechnology.

[6] Pansieri J., Gerstenmayer M., Lux F., Mériaux S., Tillement O., Forge V., Larrat B., Marquette C. (2018). Magnetic Nanoparticles Applications for Amyloidosis Study and Detection: A Review. Nanomaterials.

[7] Panahi Y., Farshbaf M., Mohammadhosseini M., Mirahadi M., Khalilov R., Saghfi S., Akbarzadeh A. (2017). Recent Advances on Liposomal Nanoparticles: Synthesis, Characterization and Biomedical Applications. Artif. Cells Nanomed. Biotechnol..

[8] Rao J.P., Geckeler K.E. (2011). Polymer Nanoparticles: Preparation Techniques and Size-Control Parameters. Prog. Polym. Sci..

[9] Liechty W.B., Kryscio D.R., Slaughter B.V., Peppas N.A. (2010). Polymers for Drug Delivery Systems. Annu. Rev. Chem. Biomol. Eng..

[10] Andrianov A.K., Payne L.G. (1998). Polymeric Carriers for Oral Uptake of Microparticulates. Adv. Drug Deliv. Rev..

[11] Rinaudo M. (2007). Main Properties and Current Applications of Some Polysaccharides as Biomaterials. Polym. Int..

[12] Sun J., Tan H. (2013). Alginate-Based Biomaterials for Regenerative Medicine Applications. Materials.

[13] Salleh S.N., Fairus A.A.H., Zahary M.N., Raj N.B., Jalil A.M.M. (2019). Unravelling the Effects of Soluble Dietary Fibre Supplementation on Energy Intake and Perceived Satiety in Healthy Adults: Evidence from Systematic Review and Meta-Analysis of Randomised-Controlled Trials. Foods.

[14] Pawar S.N., Edgar K.J. (2012). Alginate Derivatization: A Review of Chemistry, Properties and Applications. Biomaterials.

[15] Lim F., Sun A.M. (1980). Microencapsulated Islets as Bioartificial Endocrine Pancreas. Science.

[16] Paques J.P., Van Der Linden E., Van Rijn C.J.M., Sagis L.M.C. (2014). Preparation Methods of Alginate Nanoparticles. Adv. Colloid Interface Sci..

[17] Ismail I., Fauzi N.H.M., Baki M.Z., Hoon H.L. (2016). Effects of Different Drying Methods and Hydrocolloids on Quality Properties of Semi-Dried Catfish Jerky. Malays. J. Appl. Sci..

[18] Lee K.Y., Mooney D.J. (2012). Alginate: Properties and Biomedical Applications. Prog. Polym. Sci..

[19] Ching S.H., Bansal N., Bhandari B. (2017). Alginate Gel Particles—A Review of Production Techniques and Physical Properties. Crit. Rev. Food Sci. Nutr..

[20] Loquercio A., Castell-Perez E., Gomes C., Moreira R.G. (2015). Preparation of Chitosan-Alginate Nanoparticles for Trans-Cinnamaldehyde Entrapment. J. Food Sci..

[21] Spadari C.D.C., Lopes L.B., Ishida K. (2017). Potential Use of Alginate-Based Carriers as Antifungal Delivery System. Front. Microbiol..

[22] Aisyah N., Muhammad N., Hashim H., Huda N. (2017). Improving the Texture of Sardine Surimi Using Duck Feet Gelatin. J. Agrobiotechnol..

[23] Tang Y., Lan X., Liang C., Zhong Z., Xie R., Zhou Y., Miao X., Wang H., Wang W. (2019). Honey Loaded Alginate/PVA Nanofibrous Membrane as Potential Bioactive Wound Dressing. Carbohydr. Polym..

[24] Tavakoli J., Laisak E., Gao M., Tang Y. (2019). AIEgen Quantitatively Monitoring the Release of Ca2+ during Swelling and Degradation Process in Alginate Hydrogels. Mater. Sci. Eng..

[25] Al-kafaween M.A., Mohd Hilmi A.B., Jaffar N., Nagi Al-Jamal H.A., Zahri M.K., Amonov M., Mabrouka B., Elsahoryi N.A. (2020). Effects of Trigona Honey on the Gene Expression Profile of Pseudomonas Aeruginosa ATCC 10145 and Streptococcus Pyogenes ATCC 19615. Jordan J. Biol. Sci..

[26] Rehm B.H.A., Valla S. (1997). Bacterial Alginates: Biosynthesis and Applications. Appl. Microbiol. Biotechnol..

[27] Draget K.I., Smidsrød O., Skjåk-Bræk G. (2005). Alginates from Algae. Biol. Chem. Biotechnol. Appl..

[28] Simsek-Ege F.A., Bond G.M., Stringer J. (2003). Polyelectrolye Complex Formation between Alginate and Chitosan as a Function of PH. J. Appl. Polym. Sci..

[29] Skjåk-Bræk G., Murano E., Paoletti S. (1989). Alginate as Immobilization Material. II: Determination of Polyphenol Contaminants by Fluorescence Spectroscopy, and Evaluation of Methods for Their Removal. Biotechnol. Bioeng..

[30] Wijesinghe W.A.J.P., Jeon Y.J. (2012). Enzyme-Assistant Extraction (EAE) of Bioactive Components: A Useful Approach for Recovery of Industrially Important Metabolites from Seaweeds: A Review. Fitoterapia.

[31] Sharma A., Gupta M.N. (2002). Three Phase Partitioning of Carbohydrate Polymers: Separation and Purification of Alginates. Carbohydr. Polym..

[32] Gigliobianco M.R., Casadidio C., Censi R., Di Martino P. (2018). Nanocrystals of Poorly Soluble Drugs: Drug Bioavailability and Physicochemical Stability. Pharmaceutics.

[33] Hamidi M., Azadi A., Rafiei P. (2008). Hydrogel Nanoparticles in Drug Delivery. Adv. Drug Deliv. Rev..

[34] Jeevanandam J., Barhoum A., Chan Y.S., Dufresne A., Danquah M.K. (2018). Review on Nanoparticles and Nanostructured Materials: History, Sources, Toxicity and Regulations. Beilstein J. Nanotechnol..

[35] Wei Q., Tao D., Xu Y. (2012). Functional Nanofibers and Their Applications.

[36] Cai Y., Wei Q., Huang F. (2012). Processing of Composite Functional Nanofibers. Functional Nanofibers and their Applications.

[37] Al-Enizi A.M., Zagho M.M., Elzatahry A.A. (2018). Polymer-Based Electrospun Nanofibers for Biomedical Applications. Nanomaterials.

[38] Han X., Huo P., Ding Z., Kumar P., Liu B. (2019). Preparation of Lutein-Loaded PVA/Sodium Alginate Nanofibers and Investigation of Its Release Behavior. Pharmaceutics.

[39] Li W.J., Laurencin C.T., Caterson E.J., Tuan R.S., Ko F.K. (2001). Electrospun Nanofibrous Structure: A Novel Scaffold for Tissue Engineering. J. Biomed. Mater. Res..

[40] Paques J.P., Sagis L.M.C., van Rijn C.J.M., van der Linden E. (2014). Nanospheres of Alginate Prepared through w/o Emulsification and Internal Gelation with Nanoparticles of CaCO3. Food Hydrocoll..

[41] Shehata T.M., Ibrahima M.M. (2019). BÜCHI Nano Spray Dryer B-90: A Promising Technology for the Production of Metformin Hydrochloride-Loaded Alginate–Gelatin Nanoparticles. Drug Dev. Ind. Pharm..

[42] Zohri M., Alavidjeh M.S., Haririan I., Ardestani M.S., Ebrahimi S.E.S., Sani H.T., Sadjadi S.K. (2010). A Comparative Study Between the Antibacterial Effect of Nisin and Nisin-Loaded Chitosan/Alginate Nanoparticles on the Growth of Staphylococcus Aureus in Raw and Pasteurized Milk Samples. Probiotics Antimicrob. Proteins.

[43] Dai L., Zhan X., Wei Y., Sun C., Mao L., McClements D.J., Gao Y. (2018). Composite Zein—Propylene Glycol Alginate Particles Prepared Using Solvent Evaporation: Characterization and Application as Pickering Emulsion Stabilizers. Food Hydrocoll..

[44] Wang F., Yuan J., Zhang Q., Yang S., Jiang S., Huang C. (2018). PTX-Loaded Three-Layer PLGA/CS/ALG Nanoparticle Based on Layer-by-Layer Method for Cancer Therapy. J. Biomater. Sci. Polym. Ed..

[45] Daemi H., Barikani M. (2012). Synthesis and Characterization of Calcium Alginate Nanoparticles, Sodium Homopolymannuronate Salt and Its Calcium Nanoparticles. Sci. Iran..

[46] Alallam B., Altahhan S., Taher M., Mohd Nasir M.H., Doolaanea A.A. (2020). Electrosprayed Alginate Nanoparticles as CRISPR Plasmid DNA Delivery Carrier: Preparation, Optimization, and Characterization. Pharmaceuticals.

[47] Zhang J., Wang X.X., Zhang B., Ramakrishna S., Yu M., Ma J.W., Long Y.Z. (2018). In Situ Assembly of Well-Dispersed Ag Nanoparticles throughout Electrospun Alginate Nanofibers for Monitoring Human Breath—Smart Fabrics. ACS Appl. Mater. Interfaces.

[48] Jong W.H.D., Paul J.B. (2008). Drug Delivery and Nanoparticles: Applications and Hazards. Int. J. Nanomed..

[49] Dizaj S.M., Vazifehasl Z., Salatin S., Adibkia K., Javadzadeh Y. (2015). Nanosizing of Drugs: Effect on Dissolution Rate. Res. Pharm. Sci..

[50] Alshora D.H., Ibrahim M.A., Alanazi F.K. (2016). Nanotechnology from Particle Size Reduction to Enhancing Aqueous Solubility.

[51] Hassani A., Mahmood S., Enezei H.H., Hussain S.A., Hamad H.A., Aldoghachi A.F., Hagar A., Doolaanea A.A., Ibrahim W.N. (2020). Formulation, Characterization and Biological Activity Screening of Sodium Alginate-Gum Arabic Nanoparticles Loaded with Curcumin. Molecules.

[52] Ortega E., Blanco S., Ruiz A., Peinado M.Á., Peralta S., Morales M.E. (2020). Lipid nanoparticles for the transport of drugs like dopamine through the blood–brain barrier. Beilstein J. Nanotechnol..

[53] Thomas D., KurienThomas K., Latha M.S. (2020). Preparation and Evaluation of Alginate Nanoparticles Prepared by Green Method for Drug Delivery Applications. Int. J. Biol. Macromol..

[54] Roces C.B., Christensen D., Perrie Y. (2020). Translating the Fabrication of Protein-Loaded Poly(Lactic-Co-Glycolic Acid) Nanoparticles from Bench to Scale-Independent Production Using Microfluidics. Drug Deliv. Transl. Res..

[55] Ciofani G., Raffa V., Menciassi A., Dario P. (2008). Alginate and Chitosan Particles as Drug Delivery System for Cell Therapy. Biomed. Microdevices.

[56] Li M., Sun Y., Ma C., Hua Y., Zhang L., Shen J. (2020). Design and Investigation of Penetrating Mechanism of Octaarginine-Modified Alginate Nanoparticles for Improving Intestinal Insulin Delivery. J. Pharm. Sci..

[57] Alfatama M., Lim L.Y., Wong T.W. (2018). Alginate-C18 Conjugate Nanoparticles Loaded in Tripolyphosphate-Cross-Linked Chitosan-Oleic Acid Conjugate-Coated Calcium Alginate Beads as Oral Insulin Carrier. Mol. Pharm..

[58] Sorasitthiyanukarn F.N., Muangnoi C., Ratnatilaka Na Bhuket P., Rojsitthisak P., Rojsitthisak P. (2018). Chitosan/Alginate Nanoparticles as a Promising Approach for Oral Delivery of Curcumin Diglutaric Acid for Cancer Treatment. Mater. Sci. Eng..

[59] Markeb A.A., El-Maali N.A., Sayed D.M., Osama A., Abdel-Malek M.A.Y., Zaki A.H., Elwanis M.E.A., Driscoll J.J. (2016). Synthesis, Structural Characterization, and Preclinical Efficacy of a Novel Paclitaxel-Loaded Alginate Nanoparticle for Breast Cancer Treatment. Int. J. Breast Cancer.

[60] Baek S., Joo S.H., Toborek M. (2019). Treatment of Antibiotic-Resistant Bacteria by Encapsulation of ZnO Nanoparticles in an Alginate Biopolymer: Insights into Treatment Mechanisms. J. Hazard. Mater..

[61] Scolari I.R., Páez P.L., Musri M.M., Petiti J.P., Torres A., Granero G.E. (2020). Rifampicin Loaded in Alginate/Chitosan Nanoparticles as a Promising Pulmonary Carrier against Staphylococcus Aureus. Drug Deliv. Transl. Res..

[62] Holban A.M., Grumezescu A.M. (2016). Nanoarchitectonics for Smart Delivery and Drug Targeting.

[63] Uyen N.T.T., Hamid Z.A.A., Tram N.X.T., Ahmad N. (2019). Fabrication of Alginate Microspheres for Drug Delivery: A Review. Int. J. Biol. Macromol..

[64] Chan L.W., Lee H.Y., Heng P.W.S. (2006). Mechanisms of External and Internal Gelation and Their Impact on the Functions of Alginate as a Coat and Delivery System. Carbohydr. Polym..

[65] Leong J.Y., Lam W.H., Ho K.W., Voo W.P., Lee M.F.X., Lim H.P., Lim S.L., Tey B.T., Poncelet D., Chan E.S. (2016). Advances in Fabricating Spherical Alginate Hydrogels with Controlled Particle Designs by Ionotropic Gelation as Encapsulation Systems. Particuology.

[66] Pestovsky Y.S., Martínez-Antonio A. (2019). The Synthesis of Alginate Microparticles and Nanoparticles. Drug Des. Intellect. Prop. Int. J..

[67] Reis C.P., Neufeld R.J., Vilela S., Ribeiro A.J., Veiga F. (2006). Review and Current Status of Emulsion/Dispersion Technology Using an Internal Gelation Process for the Design of Alginate Particles. J. Microencapsul..

[68] Liu X.D., Yu W.Y., Zhang Y., Xue W.M., Yu W.T., Xiong Y., Ma X.J., Chen Y., Yuan Q. (2002). Characterization of Structure and Diffusion Behaviour of Ca-Alginate Beads Prepared with External or Internal Calcium Sources. J. Microencapsul..

[69] Rosch J.G., Brown A.L., Duross A.N., Duross E.L., Sahay G., Sun C. (2018). Nanoalginates via Inverse-Micelle Synthesis: Doxorubicin-Encapsulation and Breast Cancer Cytotoxicity. Nanoscale Res. Lett..

[70] Spadari C.C., de Bastiani F.W.M.D.S., Lopes L.B., Ishida K. (2019). Alginate Nanoparticles as Non-Toxic Delivery System for Miltefosine in the Treatment of Candidiasis and Cryptococcosis. Int. J. Nanomed..

[71] Quintanar-Guerrero D., Allémann E., Fessi H., Doelker E. (1999). Pseudolatex Preparation Using a Novel Emulsion-Diffusion Process Involving Direct Displacement of Partially Water-Miscible Solvents by Distillation. Int. J. Pharm..

[72] Néstor Mendoza-MuñozSergio Alcalá-AlcaláDavid Quintanar-Guerrero (2016). Preparation of Polymer Nanoparticles by the Emulsification-Solvent Evaporation Method: From Vanderhoff’s Pioneer Approach to Recent Adaptations. Polymer Nanoparticles for Nanomedicines.

[73] Muhaimin, Bodmeier R. (2017). Effect of Solvent Type on Preparation of Ethyl Cellulose Microparticles by Solvent Evaporation Method with Double Emulsion System Using Focused Beam Reflectance Measurement. Polym. Int..

[74] Lemoine D., Préat V. (1998). Polymeric Nanoparticles as Delivery System for Influenza Virus Glycoproteins. J. Control. Release.

[75] Urbaniak T., Musiał W. (2019). Influence of Solvent Evaporation Technique Parameters on Diameter of Submicron Lamivudine-Poly-ε-Caprolactone Conjugate Particles. Nanomaterials.

[76] Subedi G., Shrestha A.K., Shakya S. (2016). Study of Effect of Different Factors in Formulation of Micro and Nanospheres with Solvent Evaporation Technique. Open Pharm. Sci. J..

[77] Joshy K.S., George A., Jose J., Kalarikkal N., Pothen L.A., Thomas S. (2017). Novel Dendritic Structure of Alginate Hybrid Nanoparticles for Effective Anti-Viral Drug Delivery. Int. J. Biol. Macromol..

[78] Seyam S., Nordin N.A., Alfatama M. (2020). Recent Progress of Chitosan and Chitosan Derivatives-Based Nanoparticles: Pharmaceutical Perspectives of Oral Insulin Delivery. Pharmaceuticals.

[79] Sepúlveda-Rivas S., Fritz H.F., Valenzuela C., Santiviago C.A., Morales J.O. (2019). Development of Novel EE/Alginate Polyelectrolyte Complex Nanoparticles for Lysozyme Delivery: Physicochemical Properties and in Vitro Safety. Pharmaceutics.

[80] Rajaonarivony M., Vauthier C., Couarraze G., Puisieux F., Couvreur P. (1993). Development of a New Drug Carrier Made from Alginate. J. Pharm. Sci..

[81] Sæther H.V., Holme H.K., Maurstad G., Smidsrød O., Stokke B.T. (2008). Polyelectrolyte Complex Formation Using Alginate and Chitosan. Carbohydr. Polym..

[82] Lertsutthiwong P., Rojsitthisak P., Nimmannit U. (2009). Preparation of Turmeric Oil-Loaded Chitosan-Alginate Biopolymeric Nanocapsules. Mater. Sci. Eng..

[83] Grebinişan D., Holban M., Şunel V., Popa M., Desbrieres J., Lionte C. (2011). Novel Acyl Derivatives of N-(p-Aminobenzoyl)-l-Glutamine Encapsulated in Polymeric Nanocapsules with Potential Antitumoral Activity. Cellul. Chem. Technol..

[84] Khalid A., Bashir S., Sohail M., Amirzada M.I. (2018). Characterization of Doxorubicin Nanoparticles Prepared by Ionic Gelation. Trop. J. Pharm. Res..

[85] Katuwavila N.P., Perera A.D.L.C., Dahanayake D., Karunaratne V., Amaratunga G.A.J., Karunaratne D.N. (2016). Alginate Nanoparticles Protect Ferrous from Oxidation: Potential Iron Delivery System. Int. J. Pharm..

[86] Rahaiee S., Hashemi M., Shojaosadati S.A., Moini S., Razavi S.H. (2017). Nanoparticles Based on Crocin Loaded Chitosan-Alginate Biopolymers: Antioxidant Activities, Bioavailability and Anticancer Properties. Int. J. Biol. Macromol..

[87] Jayapal J.J., Dhanaraj S. (2017). Exemestane Loaded Alginate Nanoparticles for Cancer Treatment: Formulation and in Vitro Evaluation. Int. J. Biol. Macromol..

[88] Saralkar P., Dash A.K. (2017). Alginate Nanoparticles Containing Curcumin and Resveratrol: Preparation, Characterization, and In Vitro Evaluation Against DU145 Prostate Cancer Cell Line. AAPS PharmSciTech.

[89] Yoncheva K., Merino M., Shenol A., Daskalov N.T., Petkov P.S., Vayssilov G.N., Garrido M.J. (2019). Optimization and In-Vitro/in-Vivo Evaluation of Doxorubicin-Loaded Chitosan-Alginate Nanoparticles Using a Melanoma Mouse Model. Int. J. Pharm..

[90] Liu J., Xiao J., Li F., Shi Y., Li D., Huang Q. (2018). Chitosan-Sodium Alginate Nanoparticle as a Delivery System for ε-Polylysine: Preparation, Characterization and Antimicrobial Activity. Food Control.

[91] Bhattacharyya A., Mukherjee D., Mishra R., Kundu P.P. (2017). Preparation of Polyurethane–Alginate/Chitosan Core Shell Nanoparticles for the Purpose of Oral Insulin Delivery. Eur. Polym. J..

[92] Jardim K.V., Palomec-Garfias A.F., Andrade B.Y.G., Chaker J.A., Báo S.N., Márquez-Beltrán C., Moya S.E., Parize A.L., Sousa M.H. (2018). Novel Magneto-Responsive Nanoplatforms Based on MnFe2O4 Nanoparticles Layer-by-Layer Functionalized with Chitosan and Sodium Alginate for Magnetic Controlled Release of Curcumin. Mater. Sci. Eng..

[93] Ye S., Wang C., Liu X., Tong Z. (2005). Multilayer Nanocapsules of Polysaccharide Chitosan and Alginate through Layer-by-Layer Assembly Directly on PS Nanoparticles for Release. J. Biomater. Sci. Polym. Ed..

[94] Liu W., Liu J., Li T., Liu C., Liu W. (2013). Improved Physical and in Vitro Digestion Stability of a Polyelectrolyte Delivery System Based on Layer-by-Layer Self-Assembly Alginate-Chitosan-Coated Nanoliposomes. J. Agric. Food Chem..

[95] Ge L., Webster T.J. (2017). Doxorubicin-Loaded Poly (Lactic-Co-Glycolic Acid) Nanoparticles Coated with Chitosan/Alginate by Layer by Layer Technology for Antitumor Applications. Int. J. Nanomed..

[96] Khan M.A., Yue C., Fang Z., Hu S., Cheng H., Bakry A.M., Liang L. (2019). Alginate/Chitosan-Coated Zein Nanoparticles for the Delivery of Resveratrol. J. Food Eng..

[97] Poozesh S., Bilgili E. (2019). Scale-up of Pharmaceutical Spray Drying Using Scale-up Rules: A Review. Int. J. Pharm..

[98] O’Sullivan J.J., Norwood E.A., O’Mahony J.A., Kelly A.L. (2019). Atomisation Technologies Used in Spray Drying in the Dairy Industry: A Review. J. Food Eng..

[99] Arpagaus C., Collenberg A., Rütti D., Assadpour E., Jafari S.M. (2018). Nano Spray Drying for Encapsulation of Pharmaceuticals. Int. J. Pharm..

[100] Ziaee A., Albadarin A.B., Padrela L., Femmer T., O’Reilly E., Walker G. (2019). Spray Drying of Pharmaceuticals and Biopharmaceuticals: Critical Parameters and Experimental Process Optimization Approaches. Eur. J. Pharm. Sci..

[101] El-Missiry M.A., Othman A.I., Amer M.A., Sedki M., Ali S.M., El-Sherbiny I.M. (2020). Nanoformulated Ellagic Acid Ameliorates Pentylenetetrazol-Induced Experimental Epileptic Seizures by Modulating Oxidative Stress, Inflammatory Cytokines and Apoptosis in the Brains of Male Mice. Metab. Brain Dis..

[102] De Cicco F., Porta A., Sansone F., Aquino R.P., Del Gaudio P. (2014). Nanospray Technology for an in Situ Gelling Nanoparticulate Powder as a Wound Dressing. Int. J. Pharm..

[103] Arpagaus C. (2018). Pharmaceutical Particle Engineering via Nano Spray Drying—Process Parameters and Application Examples on the Laboratory-Scale. Int. J. Med. Nano Res..

[104] Correâ-Filho L.C., Lourenço M.M., Moldaõ-Martins M., Alves V.D. (2019). Microencapsulation of β-Carotene by Spray Drying: Effect of Wall Material Concentration and Drying Inlet Temperature. Int. J. Food Sci..

[105] Su C.Y., Wang J.C., Chen C.Y., Chu K., Lin C.K. (2019). Spherical Composite Powder by Coupling Polymethyl Methacrylate and Boron Nitride via Spray Drying for Cosmetic Application. Materials.

[106] Assadpour E., Jafari S.M. (2019). Advances in Spray-Drying Encapsulation of Food Bioactive Ingredients: From Microcapsules to Nanocapsules. Annu. Rev. Food Sci. Technol..

[107] Yuan Y., Fu A., Wang Y., Guo P., Wu G. (2017). Colloids and Surfaces A: Physicochemical and Engineering Aspects Spray Drying Assisted Assembly of ZnO Nanocrystals Using Cellulose as Sacrificial Template and Studies on Their Photoluminescent and Photocatalytic Properties. Colloids Surf. A Physicochem. Eng. Asp..

[108] Yaghoobi N., Majidi R.F., Faramarzi M.A., Baharifar H., Amani A. (2017). Preparation, Optimization and Activity Evaluation of PLGA/Streptokinase Nanoparticles Using Electrospray. Adv. Pharm. Bull..

[109] Rutkowski S., Si T., Gai M., Frueh J., He Q. (2018). Hydrodynamic Electrospray Ionization Jetting of Calcium Alginate Particles: Effect of Spray-Mode, Spraying Distance and Concentration. RSC Adv..

[110] Mehregan Nikoo A., Kadkhodaee R., Ghorani B., Razzaq H., Tucker N. (2016). Controlling the Morphology and Material Characteristics of Electrospray Generated Calcium Alginate Microhydrogels. J. Microencapsul..

[111] Correia C.R., Ghasemzadeh-Hasankolaei M., Mano J.F. (2019). Cell Encapsulation in Liquified Compartments: Protocol Optimization and Challenges. PLoS ONE.

[112] Suksamran T., Opanasopit P., Rojanarata T., Ngawhirunpat T., Ruktanonchai U., Supaphol P. (2009). Biodegradable Alginate Microparticles Developed by Electrohydrodynamic Spraying Techniques for Oral Delivery of Protein. J. Microencapsul..

[113] Naim M.N., Mokhtar M.N., Rahmam S., Bakar N.F.A., Ng E. (2016). Encapsulation of Bioactive Compound from Extracted Jasmine Flower Using β-Cyclodextrin via Electrospray. IOP Conf. Ser. Earth Environ. Sci..

[114] Torres-Chávez P.I., Ramírez-Wong B., Rangel-Vázquez N.A., Barreras-Urbina C.G., Tapia-Hernández J.A., Plascencia-Jatomea M., Rodríguez-Félix F., Rascón-Chu A. (2015). Micro- and Nanoparticles by Electrospray: Advances and Applications in Foods. J. Agric. Food Chem..

[115] Tsai S., Ting Y. (2019). Synthesize of Alginate/Chitosan Bilayer Nanocarrier by CCD-RSM Guided Co-Axial Electrospray: A Novel and Versatile Approach. Food Res. Int..

[116] Xu Y., Skotak M., Hanna M. (2006). Electrospray Encapsulation of Water-Soluble Protein with Polylactide. I. Effects of Formulations and Process on Morphology and Particle Size. J. Microencapsul..

[117] Wang C., Wang J., Zeng L., Qiao Z., Liu X., Liu H., Zhang J., Ding J. (2019). Fabrication of Electrospun Polymer Nanofibers with Diverse Morphologies. Molecules.

[118] Ignatious F., Sun L., Lee C.P., Baldoni J. (2010). Electrospun Nanofibers in Oral Drug Delivery. Pharm. Res..

[119] Chen S., Li R., Li X., Xie J. (2018). Electrospinning: An Enabling Nanotechnology Platform for Drug Delivery and Regenerative Medicine. Adv. Drug Deliv. Rev..

[120] Li W.J., Tuan R.S. (2009). Fabrication and Application of Nanofibrous Scaffolds in Tissue Engineering. Curr. Protoc. Cell Biol..

[121] Anu Bhushani J., Anandharamakrishnan C. (2014). Electrospinning and Electrospraying Techniques: Potential Food Based Applications. Trends Food Sci. Technol..

[122] Haider A., Haider S., Kang I.K. (2018). A Comprehensive Review Summarizing the Effect of Electrospinning Parameters and Potential Applications of Nanofibers in Biomedical and Biotechnology. Arab. J. Chem..

[123] Mokhena T.C., Luyt A.S. (2017). Development of Multifunctional Nano/Ultrafiltration Membrane Based on a Chitosan Thin Film on Alginate Electrospun Nanofibres. J. Clean. Prod..

[124] Ni P., Bi H., Zhao G., Han Y., Wickramaratne M.N., Dai H., Wang X. (2019). Electrospun Preparation and Biological Properties in Vitro of Polyvinyl Alcohol/Sodium Alginate/Nano-Hydroxyapatite Composite Fiber Membrane. Colloids Surf. B Biointerfaces.

[125] Guo J., Zhang Q., Cai Z., Zhao K. (2016). Preparation and Dye Filtration Property of Electrospun Polyhydroxybutyrate–Calcium Alginate/Carbon Nanotubes Composite Nanofibrous Filtration Membrane. Sep. Purif. Technol..

[126] Yeo M., Kim G.H. (2019). Nano/Microscale Topographically Designed Alginate/PCL Scaffolds for Inducing Myoblast Alignment and Myogenic Differentiation. Carbohydr. Polym..

[127] De Silva R.T., Mantilaka M.M.M.G.P.G., Goh K.L., Ratnayake S.P., Amaratunga G.A.J., De Silva K.M.N. (2017). Magnesium Oxide Nanoparticles Reinforced Electrospun Alginate-Based Nanofibrous Scaffolds with Improved Physical Properties. Int. J. Biomater..

[128] Lopes M., Abrahim B., Veiga F., Seiça R., Cabral M., Arnaud P., Andrade J.C., Ribeiro A.J., Lopes M., Abrahim B. (2016). Preparation Methods and Applications behind Alginate-Based Particles. Expert Opin. Drug Deliv..

[129] Ige O.O., Umoru L.E., Aribo S. (2012). Natural Products: A Minefield of Biomaterials. ISRN Mater. Sci..

[130] Orive G., Ponce S., Hernández R.M., Gascón A.R., Igartua M., Pedraz J.L. (2002). Biocompatibility of Microcapsules for Cell Immobilization Elaborated with Different Type of Alginates. Biomaterials.

[131] Dusseault J., Tam S.K., Ménard M., Polizu S., Jourdan G., Yahia L., Hallé J.-P. (2006). Evaluation of Alginate Purification Methods: Effect on Polyphenol, Endotoxin, and Protein Contamination. J. Biomed. Mater. Res. Part A.

[132] Flo T.H., Ryan L., Latz E., Takeuchi O., Monks B.G., Lien E., Halaas O., Akira S., Skjåk-Bræk G., Golenbock D.T. (2002). Involvement of Toll-like Receptor (TLR) 2 and TLR4 in Cell Activation by Mannuronic Acid Polymers. J. Biol. Chem..

[133] Rahaiee S., Shojaosadati S.A., Hashemi M., Moini S., Razavi S.H. (2015). Improvement of Crocin Stability by Biodegradeble Nanoparticles of Chitosan-Alginate. Int. J. Biol. Macromol..

[134] Hasnain M.S., Nayak A.K. (2020). Alginates: Versatile Polymers in Biomedical Applications and Therapeutics.

[135] Pawar S.N. (2017). Chemical Modification of Alginate. Seaweed Polysaccharides: Isolation, Biological and Biomedical Applications.

[136] Banks S.R., Enck K., Wright M., Opara E.C., Welker M.E. (2019). Chemical Modification of Alginate for Controlled Oral Drug Delivery. J. Agric. Food Chem..

[137] Nataraj D., Reddy N. (2019). Chemical modifications of alginate and its derivatives. Int. J. Chem. Res..

[138] Pawar S.N., Edgar K.J. (2011). Chemical Modification of Alginates in Organic Solvent Systems. Biomacromolecules.

[139] Yasmin F., Chen X., Eames B.F. (2019). Effect of Process Parameters on the Initial Burst Release of Protein-Loaded Alginate Nanospheres. J. Funct. Biomater..

[140] Sarei F., Dounighi N., Zolfagharian H., Khaki P., Bidhendi S. (2013). Alginate Nanoparticles as a Promising Adjuvant and Vaccine Delivery System. Indian J. Pharm. Sci..

[141] Mokhtari S., Jafari S.M., Assadpour E. (2017). Development of a Nutraceutical Nano-Delivery System through Emulsification/Internal Gelation of Alginate. Food Chem..

[142] Lokhande A.B., Mishra S., Kulkarni R.D., Naik J.B. (2013). Influence of Different Viscosity Grade Ethylcellulose Polymers on Encapsulation and in Vitro Release Study of Drug Loaded Nanoparticles. J. Pharm. Res..

[143] Lee J.H., Yeo Y. (2015). Controlled Drug Release from Pharmaceutical Nanocarriers. Chem. Eng. Sci..

[144] Ahdyani R., Novitasari L., Martien R., Danarti R. (2019). Formulation and Characterization of Timolol Maleate-Loaded Nanoparticles Gel by Ionic Gelation Method Using Chitosan and Sodium Alginate. Int. J. Appl. Pharm..

[145] Zimet P., Mombrú Á.W., Faccio R., Brugnini G., Miraballes I., Rufo C., Pardo H. (2018). Optimization and Characterization of Nisin-Loaded Alginate-Chitosan Nanoparticles with Antimicrobial Activity in Lean Beef. LWT Food Sci. Technol..

[146] Mansourpour M., Mahjub R., Amini M., Ostad S.N., Shamsa E.S., Rafiee-Tehrani M., Dorkoosh F.A. (2015). Development of Acid-Resistant Alginate/Trimethyl Chitosan Nanoparticles Containing Cationic β-Cyclodextrin Polymers for Insulin Oral Delivery. AAPS PharmSciTech.

[147] Govindaraju R., Karki R., Chandrashekarappa J., Santhanam M., Shankar A.K.K., Joshi H.K., Divakar G. (2019). Enhanced Water Dispersibility of Curcumin Encapsulated in Alginate-Polysorbate 80 Nano Particles and Bioavailability in Healthy Human Volunteers. Pharm. Nanotechnol..

[148] Baghbani F., Moztarzadeh F., Mohandesi J.A., Yazdian F., Mokhtari-Dizaji M., Hamedi S. (2016). Formulation Design, Preparation and Characterization of Multifunctional Alginate Stabilized Nanodroplets. Int. J. Biol. Macromol..

[149] Elgegren M., Kim S., Cordova D., Silva C., Noro J., Cavaco-Paulo A., Nakamatsu J. (2019). Ultrasound-Assisted Encapsulation of Sacha Inchi (Plukenetia Volubilis Linneo.) Oil in Alginate-Chitosan Nanoparticles. Polymers.

[150] Scolari I.R., Páez P.L., Sánchez-Borzone M.E., Granero G.E. (2019). Promising Chitosan-Coated Alginate-Tween 80 Nanoparticles as Rifampicin Coadministered Ascorbic Acid Delivery Carrier Against Mycobacterium Tuberculosis. AAPS PharmSciTech.

[151] Rosa G.D., Iommelli R., La Rotonda M.I., Miro A., Quaglia F. (2000). Influence of the Co-Encapsulation of Different Non-Ionic Surfactants on the Properties of PLGA Insulin-Loaded Microspheres. J. Control. Release.

[152] Sabbagh H.A.K., Hussein-Al-Ali S.H., Hussein M.Z., Abudayeh Z., Ayoub R., Abudoleh S.M. (2020). A Statistical Study on the Development of Metronidazole-Chitosan-Alginate Nanocomposite Formulation Using the Full Factorial Design. Polymers.

[153] Cafaggi S., Russo E., Stefani R., Leardi R., Caviglioli G., Parodi B., Bignardi G., De Totero D., Aiello C., Viale M. (2007). Preparation and Evaluation of Nanoparticles Made of Chitosan or N-Trimethyl Chitosan and a Cisplatin-Alginate Complex. J. Control. Release.

[154] Mane S., Ponrathnam S., Chavan N. (2016). Effect of Chemical Crosslinking on Properties of Polymer Microbeads: A Review. Can. Chem. Trans..

[155] Güncüm E., Işıklan N., Anlaş C., Ünal N., Bulut E., Bakırel T. (2018). Development and Characterization of Polymeric-Based Nanoparticles for Sustained Release of Amoxicillin—An Antimicrobial Drug. Artif. Cells Nanomed. Biotechnol..

[156] Jin M., Zheng Y., Hu Q. (2009). Preparation and Characterization of Bovine Serum Albumin Alginate/Chitosan Microspheres for Oral Administration. Asian J. Pharm. Sci..

[157] Patil S.B., Sawant K.K. (2009). Development, Optimization and in Vitro Evaluation of Alginate Mucoadhesive Microspheres of Carvedilol for Nasal Delivery. J. Microencapsul..

[158] Peretz S., Florea-Spiroiu M., Anghel D.-F., Bala D., Stoian C., Zgherea G. (2013). Preparation of Porous Calcium Alginate Beads and Their Use for Adsorption of O-Nitrophenol from Aqueous Solutions. Micro Nanoeng..

[159] Rastogi R., Sultana Y., Aqil M., Ali A., Kumar S., Chuttani K., Mishra A.K. (2007). Alginate Microspheres of Isoniazid for Oral Sustained Drug Delivery. Int. J. Pharm..

[160] Chopra M., Kaur P., Bernela M., Thakur R. (2012). Synthesis And Optimization of Streptomycin Loaded Chitosan-Alginate Nanoparticles. Int. J. Sci. Technol. Res..

[161] Lopes M.A., Abrahim-Vieira B., Oliveira C., Fonte P., Souza A.M., Lira T., Sequeira J.A., Rodrigues C.R., Cabral L.M., Sarmento B. (2015). Probing Insulin Bioactivity in Oral Nanoparticles Produced by Ultrasonication-Assisted Emulsification/Internal Gelation. Int. J. Nanomedicine.

[162] Pradhan S., Hedberg J., Blomberg E., Wold S., Odnevall Wallinder I. (2016). Effect of Sonication on Particle Dispersion, Administered Dose and Metal Release of Non-Functionalized, Non-Inert Metal Nanoparticles. J. Nanoparticle Res..

[163] Sarmento B., Ferreira D., Veiga F., Ribeiro A. (2006). Characterization of Insulin-Loaded Alginate Nanoparticles Produced by Ionotropic Pre-Gelation through DSC and FTIR Studies. Carbohydr. Polym..

[164] Venkatesan J., Bhatnagar I., Kim S.K. (2014). Chitosan-Alginate Biocomposite Containing Fucoidan for Bone Tissue Engineering. Mar. Drugs.

[165] Emami J., Shetab Boushehri M.S., Varshosaz J. (2014). Preparation, Characterization and Optimization of Glipizide Controlled Release Nanoparticles. Res. Pharm. Sci..

[166] Gupta V.K., Karar P.K. (2011). Optimization of Process Variables for the Preparation of Chitosanalginate Nanoparticles. Int. J. Pharm. Pharm. Sci..

[167] Samprasit W., Akkaramongkolporn P., Jaewjira S., Opanasopit P. (2018). Design of Alpha Mangostin-Loaded Chitosan/Alginate Controlled-Release Nanoparticles Using Genipin as Crosslinker. J. Drug Deliv. Sci. Technol..

[168] Nait Mohamed F.A., Laraba-Djebari F. (2016). Development and Characterization of a New Carrier for Vaccine Delivery Based on Calcium-Alginate Nanoparticles: Safe Immunoprotective Approach against Scorpion Envenoming. Vaccine.

[169] Sansdrap P., Moës A.J. (1993). Influence of Manufacturing Parameters on the Size Characteristics and the Release Profiles of Nifedipine from Poly(DL-Lactide-Co-Glycolide) Microspheres. Int. J. Pharm..

[170] Mateovic T., Kriznar B., Bogataj M., Mrhar A. (2002). The Influence of Stirring Rate on Biopharmaceutical Properties of Eudragit RS Microspheres. J. Microencapsul..

[171] Denkbas E.B., Kilic E., Birlikseven C., Ozturk E. (2002). Magnetic Chitosan Microspheres: Preparation and Characterization. React. Funct. Polym..

[172] Denkbaş E.B., Odabaşi M. (2000). Chitosan Microspheres and Sponges: Preparation and Characterization. J. Appl. Polym. Sci..

[173] Seo J.Y., Lee B., Kang T.W., Noh J.H., Kim M.J., Ji Y.B., Ju H.J., Min B.H., Kim M.S. (2018). Electrostatically Interactive Injectable Hydrogels for Drug Delivery. Tissue Eng. Regen. Med..

[174] Gierszewska M., Ostrowska-Czubenko J., Chrzanowska E. (2018). PH-Responsive Chitosan/Alginate Polyelectrolyte Complex Membranes Reinforced by Tripolyphosphate. Eur. Polym. J..

[175] Zhang G.Q., Zha L.S., Zhou M.H., Ma J.H., Liang B.R. (2005). Preparation and Characterization of PH- and Temperature-Responsive Semi-Interpenetrating Polymer Network Hydrogels Based on Linear Sodium Alginate and Crosslinked Poly(N-Isopropylacrylamide). J. Appl. Polym. Sci..

[176] Wang A., Li P., Dai Y., Zhang J., Wang A., Wei Q. (2008). Chitosan-Alginate Nanoparticles as a Novel Drug Delivery System for Nifedipine. Int. J. Biomed. Sci..

[177] Sorasitthiyanukarn F.N., Ratnatilaka Na Bhuket P., Muangnoi C., Rojsitthisak P., Rojsitthisak P. (2015). Chitosan/Alginate Nanoparticles as a Promising Carrier of Novel Curcumin Diethyl Diglutarate. Int. J. Biol. Macromol..

[178] Dupuy B., Arien A., Perrot Minnot A. (1994). FT-IR of Membranes Made with Alginate/Polylysine Complexes. Variations with the Mannuronic or Guluronic Content of the Polysaccharides. Artif. Cells Blood Substitutes Biotechnol..

[179] Gazori T., Khoshayand M.R., Azizi E., Yazdizade P., Nomani A., Haririan I. (2009). Evaluation of Alginate/Chitosan Nanoparticles as Antisense Delivery Vector: Formulation, Optimization and in Vitro Characterization. Carbohydr. Polym..

[180] Bhunchu S., Muangnoi C., Rojsitthisak P., Rojsitthisak P. (2016). Curcumin Diethyl Disuccinate Encapsulated in Chitosan/Alginate Nanoparticles for Improvement of Its in Vitro Cytotoxicity against MDA-MB-231 Human Breast Cancer Cells. Pharmazie.

[181] Thai H., Thuy Nguyen C., Thi Thach L., Thi Tran M., Duc Mai H., Thi Thu Nguyen T., Duc Le G., Van Can M., Dai Tran L., Long Bach G. (2020). Characterization of Chitosan/Alginate/Lovastatin Nanoparticles and Investigation of Their Toxic Effects in Vitro and in Vivo. Sci. Rep..

[182] Wu Y., Duong A., Lee L.J., Wyslouzil B.E. (2012). Electrospray Production of Nanoparticles for Drug/Nucleic Acid Delivery. The Delivery of Nanoparticles.

[183] Arthanari S., Mani G., Jang J.H., Choi J.O., Cho Y.H., Lee J.H., Cha S.E., Oh H.S., Kwon D.H., Jang H.T. (2014). Preparation and Characterization of Gatifloxacin-Loaded Alginate/Poly (Vinyl Alcohol) Electrospun Nanofibers. Artif. Cells Nanomed. Biotechnol..

